# Mapping heat tolerance QTLs in *Triticum durum-Aegilops speltoides* backcross introgression lines to enhance thermotolerance in wheat

**DOI:** 10.3389/fpls.2024.1485914

**Published:** 2024-12-20

**Authors:** Navaneetha Krishnan J., Satinder Kaur, Uttam Kumar, Rohtas Singh, Guriqbal Singh Dhillon, Pradeep Kumar Bhati, Parveen Chhuneja

**Affiliations:** ^1^ School of Agricultural Biotechnology, Punjab Agricultural University, Ludhiana, India; ^2^ Borlaug Institute for South Asia, Ludhiana, India; ^3^ Astralyn Agro One Person Company (OPC) Pvt. Ltd, Shamli, India

**Keywords:** heat tolerance, wheat, Aegilops speltoides, QTL mapping, Triticum durum, backcross introgression lines

## Abstract

Wheat, a major cereal crop, is the most consumed staple food after rice in India. Frequent episodes of heat waves during the past decade have raised concerns about food security under impending global warming and necessitate the development of heat-tolerant wheat cultivars. Wild relatives of crop plants serve as untapped reservoirs of novel genetic variations. In the present study a mapping population comprising 311 BC_2_F_10_ backcross introgression lines (BILs) developed by crossing *Triticum durum* and heat-tolerant diploid wild wheat relative *Aegilops speltoides* accession pau3809 was used to map QTLs for terminal heat tolerance. The homozygous BILs were evaluated for heat stress tolerance component traits under an optimum environment (OE) and a heat-stressed environment (HE) for the two cropping seasons. Data on spike length, spikelet number per spike, peduncle length, thousand-grain weight, grains per spike, days to heading, days to maturity, grain filling duration, NDVI at heading, plant height and plot yield were recorded. Genotyping-by-sequencing (GBS) of the BILs was carried out, and 2945 high-quality, polymorphic SNPs were obtained. Thirty QTLs were detected for various heat tolerance component traits on chromosomes 1A, IB, 2A, 2B, 3B, 4B, 5A, 5B, 6A and 6B with phenotypic variance ranging from 5 to 11.5%. Several candidate genes reported to play a role in heat stress responses were identified by browsing the 1.85 Mb physical region flanking the stable QTLs detected under the HE. Identified QTL and linked markers can be employed for genomics-assisted breeding for heat tolerance in wheat.

## 1 Introduction

Wheat is a major staple food cultivated across the globe and provides 20% of the dietary energy and protein for global population (Curtis et al., 2002; Shiferaw et al., 2013). The developing and developed countries contribute equally to global wheat production (Shiferaw et al., 2013). After rice, wheat is the predominant staple diet of the Indian population and is the chief ingredient of a variety of processed foods (Khatkar et al., 2016). India occupies second rank among the wheat-producing countries with a production of 110.5 million tonnes and a productivity of 3.47 tonnes/ha (Government of India (GOI), 2023). Wheat production is under constant threat due to several factors including decreasing water resources, soil fertility loss, emerging pests and diseases and global warming (Ramadas et al., 2020; Asseng et al., 2015).

Climate change poses significant risks to wheat production in the form of frequent episodes of heat waves, drought and expansion of soil salinity (Joshi and Kar, 2009; Tripathi et al., 2016; Mondal et al., 2021). High-temperature stress is emerging as a serious threat to wheat productivity and affects nearly 40% of the irrigated wheat crop globally (Iqbal et al., 2017; Farhad et al., 2023). Heat stress at the reproductive stage impairs grain-filling and seed set, resulting in reduced grain yield (Hays et al., 2007). The Indo-Gangetic plains zone, which contributes 15% to global wheat production, is more prone to climatic shifts, and it is predicted that by the end of 2050, 51% of its total area will be reclassified as a heat-stressed environment with a shortened growth season (Ortiz et al., 2008). Lobell et al. (2011) reported that during the 1980-2006 period, wheat yields were reduced up to 5.5% as a result of rising temperatures. Various metabolic processes are implicated during heat stress resulting in decreased grain weight, shriveled grains, early senescence, altered starch-lipid composition in grains, reduced starch accumulation, reduced seed germination and loss of vigour (Balla et al., 2012). Therefore, breeding for heat tolerance has gained priority in the national and international wheat improvement programmes. Heat tolerance *per se* is not a Mendelian trait, as it comprises different individual components that interact with each other to manifest heat tolerance. Several morphological, physiological, biochemical and yield-related traits are involved in conferring or expressing heat tolerance in a plant. In the case of wheat, these include and are not limited to stay green, biomass, canopy temperature, membrane thermostability, early ground cover, grain weight, grain number, tiller number, photosynthetic efficiency, production of reactive oxygen species (ROS), antioxidant enzymes and chlorophyll content (Sundeep et al., 2013; Asthir, 2015; Narayanan, 2018).

Developing breeding populations utilizing diverse genetic resources and characterizing them both phenotypically and genotypically can lead to the delineation of the genomic regions (QTL) associated with heat tolerance (Kim et al., 2017; Peverelli and Rogers, 2013). With the availability of the whole genome sequence of wheat and the recent expansion in genomics and bioinformatics tools, wheat breeding has increased its pace (Appels et al., 2018; Rasheed and Xia, 2019; Adamski et al., 2020). QTLs for heat tolerance-related traits have been reported in all 21 wheat chromosomes (Bhusal et al., 2017). There are limited reports of mapping QTLs introgressed from wild species for heat stress tolerance in wheat (Awlachew et al., 2016).

The genetic diversity for heat stress tolerance in the cultivated wheat germplasm is limited. Wild species of wheat have immensely contributed to wheat improvement in terms of being reservoirs of genes/QTLs for various biotic and abiotic stresses (Gill et al., 2006; Schneider et al., 2008; Pradhan et al., 2012; Kishii, 2019; Crespo-Herrera et al., 2019). *Aegilops speltoides* has been identified as a promising germplasm source for heat stress tolerance traits (Ehdaie and Waines, 1992; Waines, 1994; Khanna-Chopra and Viswanathan, 1999; Pradhan et al., 2012; Awlachew et al., 2016). Homeologous pairing in wheat is inhibited by the *Ph1* locus present in the long arm of the 5B chromosome (Okamoto, 1957; Sears and Okamoto, 1958; Riley and Chapman, 1958; Bhullar et al., 2014). *Ae. speltoides*, the probable donor of the wheat B genome, has genes that are epistatic to the *Ph1* locus, and these genes, called *Ph1* suppressors, were mapped to the 3S and 7S chromosomes by Dvorak et al. (2006). The degree of suppression varies among the different accessions of *Ae. speltoides*, and accordingly, they are categorized as strong, moderate or weak suppressors of the *Ph1* locus (Millet, 2008). Accessions of *Ae. speltoides* having moderate suppression activity of the *Ph1* locus can aid in the transfer of useful variability to cultivated wheat with minimal meiotic anomalies (Millet, 2008).

We had earlier reported the development of a set of 90 tetraploid genome-wide homozygous backcross introgression lines involving *T. durum* cv. PDW274 and a heat-tolerant accession of *Ae. speltoides* (Awlachew et al., 2016). We carried out introgression profiling of the BILs with 152 polymorphic SSR markers and mapped QTLs for heat tolerance component traits (Awlachew et al., 2016). For deep diving into the genome of *T. durum-Ae.speltoides*, backcross introgression lines for heat tolerance genes/QTL, we expanded the introgression panel and performed genotyping-by-sequencing to increase the resolution of QTL mapping. Identification and mapping of heat tolerance QTL using genome-wide SNP markers is being discussed in the present manuscript.

## 2 Materials and methods

### 2.1 Development of the plant material

**Figure 1:**
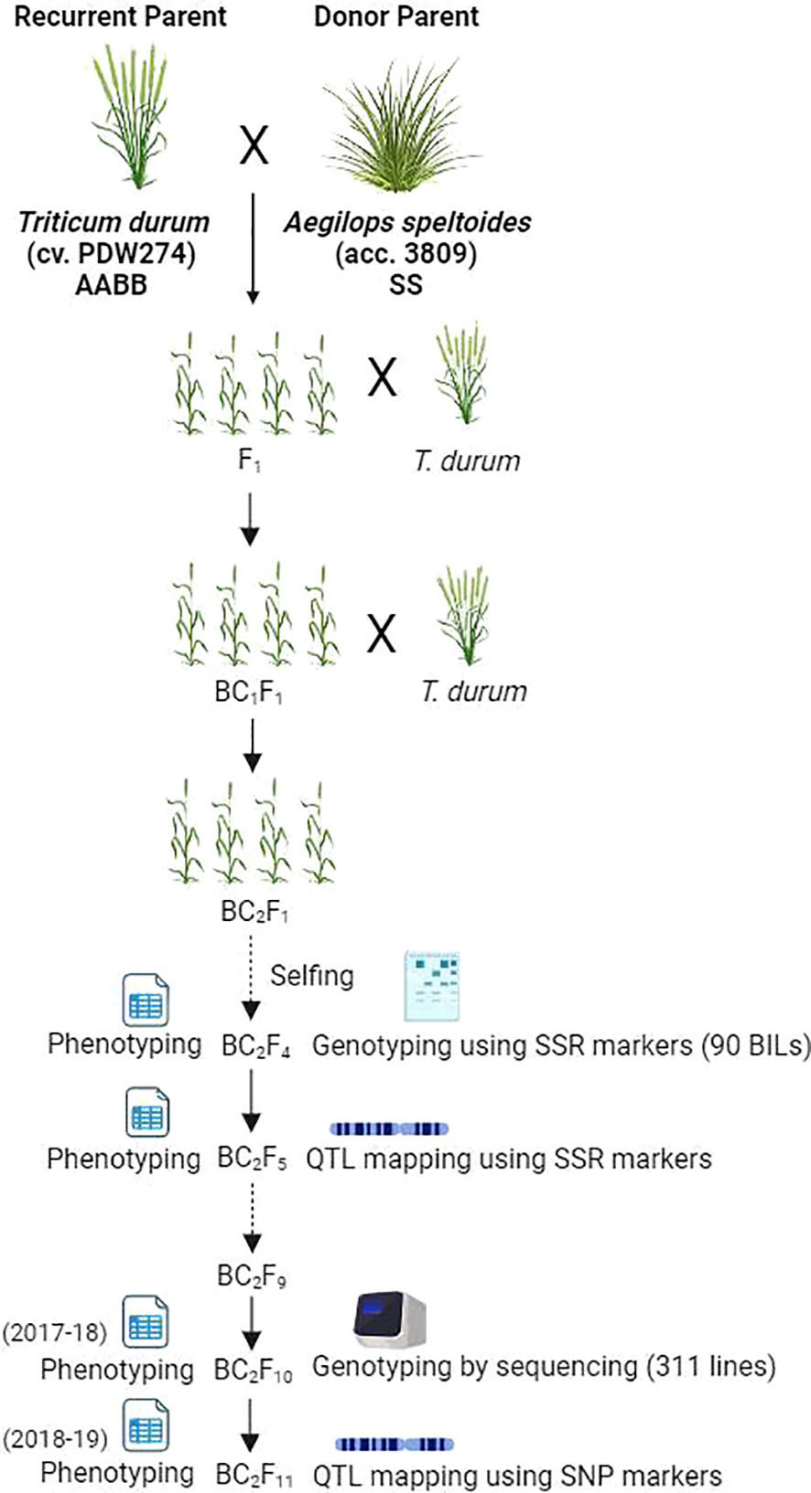
Schematic representation of the study design (created with BioRender.com).

A heat-tolerant accession of *Ae. speltoides* (2n=2x=14, SS) designated as accession pau3809 was crossed with *T. durum* cv. PDW274 (2n=4x=28, AABB) as the female parent. The F_1_ plants displayed profuse tillering and were extensively backcrossed with PDW274. Five BC_1_F_1_ plants were obtained after pollinating more than 1500 florets. The five BC_1_F_1_ plants were again backcrossed with the female parent to generate 128 BC_2_F_1_s (Awlachew et al., 2016). All the BC_2_F_1_s were selfed, and a single seed descent strategy was employed to develop 311 BC_2_F_10_ homozygous BILs. The crossing strategy adopted for generating the plant material is schematically depicted in **Figure 1**.

### 2.2 Field evaluation for heat stress tolerance

The BILs and the recurrent parent PDW274 were evaluated at the experimental farm, Borlaug Institute for South Asia (BISA), Ladhowal, Punjab, for two consecutive crop seasons(2017-18 and 2018-19). The BILs were sown on two different dates: first during the third week of November, referred to as the optimum environment (OE) and second, during the third week of December, referred to as the heat-stressed environment (HE) in a randomized block design with two replications. The OE and HE of the 2017-18 cropping season will be referred to as OE1 and HE1, and the OE and HE of the 2018-19 cropping season will be referred to as OE2 and HE2 in the subsequent sections. The details of the sowing dates are given in **Table 1**. Sowing was carried out using a limit plot planter in four-row plots of size 2.6 x 0.88m. Standard crop husbandry practices were followed to ensure a healthy crop. An aerial view of the experimental field trials is shown in **Supplementary Figure S1**. The Field Book app, an Android-based open-source application developed by Rife and Poland (2014), was used for recording the phenotypic data of all the traits except plot yield, thousand-grain weight and grains per spike. Since we delayed the sowing by a month in the case of the HE trials, we believe that the BILs experienced significant exposure to heat stress at the reproductive stage, except for very few lines that flowered too early.

**Table 1:** Sowing dates, harvesting dates, temperature and rainfall data for the two-year trials of the *T. durum-Ae. speltoides* backcross introgression lines.

Year	Sowing date		Harvesting date		Mean maximum temperature at grain filling stage		Mean minimum temperature at grain filling stage		Total rainfall received (mm)
Environment	Optimum	Heat-stressed	Optimum	Heat-stressed	Optimum	Heat-stressed	Optimum	Heat-stressed	Across the crop season
2017-18	23-11-2017	20-12-2017	29-04-2018	09-05-2018	30.5	33.7	15.6	17.7	125.4
2018-19	16-11-2018	20-12-2018	06-05-2019	15-05-2019	28.0	32.7	13.7	17.2	253.9

Data on the following phenotypic traits were recorded during the two cropping seasons: Days to heading (DTH) was calculated on a plot basis as the number of days required for the completion of spike exertion [Zadoks’ growth stage (GS) 58] of at least 50% of the plot’s vegetation cover. Days to maturity (DTM) was calculated on a plot basis as the number of days required for attaining physiological maturity (GS91) of at least 75% of the plot’s vegetation cover. For grain-filling duration (GFD), the period between the days to heading (GS58) and the completion of physiological maturity (GS91) was noted and expressed in days. Five mature primary spikes were selected at random from each plot, and the mean spikelet number per spike was calculated and recorded as spikelet number per spike (SN). The selected spikes were threshed, and the average count of the grains was recorded as grain number per spike (GPS). Spike length (SL) was calculated as the average length of five randomly selected mature primary spikes from each plot. For peduncle length (PL), the average length of peduncles corresponding to five randomly selected primary spikes was measured at physiological maturity. Plot yield (PY) was recorded as the grain yield of individual plots. Normalized difference vegetation index at heading (NDVI_H) was measured using a handheld ‘Greenseeker’ device (Trimble, USA) at the heading stage (GS 58). One reading per plot was recorded by passing the Greenseeker at 50-60 cm above the plot surface for 4-5 seconds. The device was passed over the middle of the plot canopy, excluding the exterior rows. The thousand-grain weight (TGW) was estimated using a novel image analysis-based method reported in our earlier publication (Krishnan et al., 2023).

### 2.3 Weather data

Weather data was recorded for the entire wheat growing season of 2017-18 and 2018-19 using the automatic weather data recorder at BISA, Ladhowal. Data on daily minimum and maximum temperature and rainfall were collected.

### 2.4 Statistical analysis of the phenotypic data

The best linear unbiased predictors (BLUPs) of the phenotypic values were calculated for the optimum and heat-stressed environments of both cropping seasons using the META-R software (Alvarado et al., 2020). The BLUPs were calculated for individual years and across the years for both environments, respectively. Summary statistics were computed from the BLUPs using the PAST 4.03 software (Hammer et al., 2001). The trait distribution, plotting of weather data, and correlation analysis were carried out using the ‘ggplot2’ and ‘corrplot’ packages in the R software (R Core Team, 2013; Wickham, 2016; Wei and Simko, 2021). For correlation analysis, BLUPs of the phenotypic traits over the years (pooled data) were used. Levene’s test was used to test for the equality of variances for the year-wise phenotypic data using the ‘Proc GLM’ procedure in SAS 9.4 (SAS Institute Inc, 2013). The analysis of variance for the two-year phenotypic data was carried out using SAS 9.4 (SAS Institute Inc, 2013).

### 2.5 Genotyping and introgression profiling

DNA was extracted from the BILs, the recurrent parent PDW274 and the donor parent *Ae. speltoides* acc. pau3809 using the protocol developed by Allen et al. (2006). The sequencing of the BILs was carried out using the two-enzyme genotyping-by-sequencing (GBS) approach described by Poland et al. (2012). The raw sequence files were processed using the GBS pipeline version 5.2.31 in the TASSEL software (Glaubitz et al., 2014). The SNP calling was done against the A and B genomes of the wheat genome assembly Refseq v1.0. The generated vcf file was filtered for a minimum read depth of 3 (DP3) and converted into hapmap format, resulting in 1,15,709 SNPs. The hapmap file was further filtered to identify homozygous SNPs for each parental line, and SNPs that were polymorphic between the two parents were retained that reduced the number to 10,092 SNPs. Loci with very low coverage (<50%) and high heterozygosity (>20%) were filtered out, and BILs containing more than 10% missing data were excluded. Finally, we ended up with 227 BILs with high-quality SNP data, comprising 2945 polymorphic SNPs that was used for QTL mapping. Introgression profiling was carried out using the GGT2 software (Van Berloo, 2008) to visualize the donor introgressions.

### 2.6 QTL mapping

The BLUPs of the two-year phenotypic data, pooled phenotypic data and genotypic data of the 227 BILs and the recurrent parent were used for QTL mapping in QTL IciMapping version 4.2 (Meng et al., 2015). The mapping was carried out using the likelihood ratio test based on stepwise regression for the additive QTL (RSTEP-LRT-ADD) method. The logarithm of odds (LOD) significance was fixed at 2.5. QTLs with a LOD greater than 2.5 and phenotypic variance explained (PVE) above 5% were considered significant.

### 2.7 QTL nomenclature

QTL names were assigned based on the guidelines of the International Rules of Genetic Nomenclature (Boden et al., 2023). QTLs were named in the following manner: “Q” denotes “QTL”, followed by abbreviations of the phenotypic traits (PY, TGW, GPS, SN, SL, PL, DTH, DTM, GFD and NDVI_H); “pau” stands for Punjab Agricultural University; “Td” denotes that the QTL donor parent is *T. durum*; and “As” denotes that the QTL is donated by *Ae. speltoides*; “OE” and “HE” are the abbreviations for optimum and heat-stressed environments; the last is the wheat chromosome number; if there are more than one QTL detected on a chromosome, it is numbered as 1,2,3, etc., based on their physical position.

### 2.8 Testing the allelic effect of the SNPs associated with the stable QTLs

The effect of the allelic substitution at the SNP locus associated with the stable QTLs detected in the heat-stressed environment was evaluated using the Mann-Whitney U test. The genotypes were divided into two groups based on their SNP allelic pattern. The pooled BLUPs of the two-year phenotypic data were used to generate the box plots, and the two groups were compared to test for significant median differences following the Mann-Whitney U test. Since the phenotypic data of the two allelic groups were not normally distributed, we could not proceed with the parametric tests for mean comparison. The box plots and statistics were generated using the ‘ggbetweenstats’ function of the ‘ggstatsplot’ R-package (Patil, 2021).

### 2.9 Postulation of candidate genes

The pairwise linkage disequilibrium between the SNP loci at the genome level with no missing data was calculated in Tassel v 5.0 and plotted by computing the r^2^ estimators between all pairs of SNP markers using the R software. LD decay was estimated using a spline that was fitted on a LD_½,95_, the distance at which the short-range LD is halved when using the 95% percentile of r^2^ at short range, as an estimator for LD. The chromosome-wise linkage disequilibrium (LD) decay value (in Mb) was used to define the confidence intervals to locate candidate genes. The physical interval flanking the consistent QTLs was mined for putative candidate genes based on the list of high-confidence genes in the IWGSC RefSeq v1.0 genome assembly. The web-based genome browser ‘Persephonesoft’ (https://web.persephonesoft.com/) was used for fetching the gene IDs of high-confidence genes. The functional annotations of the high-confidence genes were retrieved from https://urgi.versailles.inra.fr/download/iwgsc/IWGSC_RefSeq_Annotations/v1.0/.

## 3 Results

### 3.1 Phenotyping for heat stress tolerance component traits

**Figure 2:**
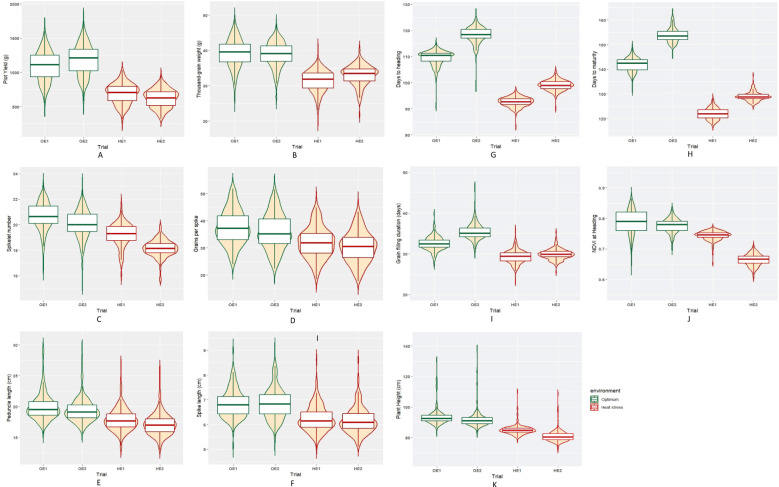
Box plots representing the distribution of various phenotypic traits under optimum and heat-stressed environments in the *T. durum*-*Ae. speltoides* BILs. **(A)** Plot yield, **(B)** Thousand-grain weight, **(C)** Spikelet number per spike, **(D)** Grains per spike, **(E)** Spike length, **(F)** Peduncle length, **(G)** Days to heading, **(H)** Days to maturity, **(I)** Grain filling duration, **(J)** NDVI at heading and **(K)** Plant height.

The summary statistics of the heat tolerance component traits are enlisted in **Table 2**, and it is evident from the mean statistics that heat stress had a negative effect on the agronomic performance of the BILs in both cropping seasons. The BILs displayed considerable variation for all the traits studied under both OE and HE and the relative distribution of various phenotypic traits under both environments is explained graphically in the form of boxplots overlaid with violin plots (**Figure 2**). The variations observed for spike and peduncle traits among the BILs and the parents are presented in **Supplementary Figures S2**, **S3**, respectively. Regarding the temperature data during the grain filling period, there was a difference of 3° to 4°C in the mean maximum and minimum temperatures between the optimum and heat-stressed environment trials (**Table 1**; **Supplementary Figure S4**). This validates the incidence of heat stress during the reproductive phase of the HE trial, and the delay of the sowing date was effective in imposing terminal heat stress. In the HE2 trial, there was a greater decline in the plot yield and NDVI of the BILs in comparison to the HE1 trial due to the high rainfall received during the January and February months (**Supplementary Figure S5**). As the late-sown BILs were in the early vegetative stage, there was a reduction in their growth and development due to waterlogging stress. The plant density of many plots declined as some proportion of the plants succumbed to the stress, leading to a decrease in the plot yield and NDVI.

**Table 2:** Summary statistics for the optimum and heat-stressed environment trials of *T. durum-Ae. speltoides* backcross introgression lines.

Statistics	Environment	PY (g)	TGW (g)	GPS	SN	SL (cm)	PL (cm)	DTH (days)	DTM (days)	GFD (days)	NDVI_H	PH (cm)
Mean	OE1	1098.01	39.33	37.58	20.60	6.83	19.93	109.55	142.04	32.49	0.79	93.54
	HE1	691.91	31.48	31.87	19.24	6.23	17.87	92.65	122.02	29.37	0.74	85.05
	OE2	1186.53	38.88	36.01	20.03	6.85	19.43	118.38	153.86	35.47	0.78	92.39
	HE2	621.93	33.01	30.30	18.13	6.15	17.16	99.02	129.04	30.02	0.66	81.00
Minimum	OE1	522.00	25.26	23.45	15.67	4.95	15.35	90.00	131.00	26.50	0.65	82.97
	HE1	246.50	19.29	17.90	15.35	4.90	12.85	81.50	116.50	22.00	0.48	78.13
	OE2	552.00	25.45	22.00	14.33	5.07	15.35	98.00	146.00	29.50	0.70	82.68
	HE2	287.00	21.12	17.30	14.67	4.98	12.75	89.00	124.00	21.50	0.52	72.20
Maximum	OE1	1629.00	49.26	52.40	23.67	9.25	30.25	115.50	149.50	41.00	0.87	130.76
	HE1	1053.00	41.14	48.35	22.18	8.90	27.32	98.00	129.00	37.50	0.83	110.15
	OE2	1773.50	47.89	51.90	23.83	9.25	30.15	126.50	163.00	48.00	0.83	138.10
	HE2	986.50	40.90	46.25	20.67	8.92	26.67	105.50	137.00	38.00	0.78	108.71
Standard deviation	OE1	225.65	4.16	6.44	1.33	0.61	2.27	2.96	3.05	2.00	0.04	5.68
	HE1	155.09	3.21	5.91	1.09	0.58	1.93	2.15	2.66	1.98	0.04	3.21
	OE2	235.20	3.73	6.64	1.41	0.69	2.15	3.20	2.99	2.21	0.02	6.72
	HE2	143.47	2.98	5.86	0.93	0.60	1.99	2.19	2.42	2.18	0.05	4.09
Coefficient of Variation	OE1	20.55	10.57	17.13	6.45	8.95	11.38	2.70	2.14	6.15	5.12	6.07
	HE1	22.41	10.19	18.53	5.65	9.26	10.80	2.32	2.18	6.73	5.73	3.77
	OE2	19.82	9.60	18.42	7.03	10.09	11.08	2.71	1.95	6.24	2.95	7.27
	HE2	23.06	9.03	19.35	5.13	9.50	11.60	2.18	1.87	7.25	7.32	5.05

PY, Plot yield; TGW, Thousand-grain weight; GPS, Grains per spike; SN, Spikelet number per spike; SL, Spike length; PL, Peduncle length; DTH, Days to heading; DTM, Days to maturity; GFD, Grain filling duration; NDVI_H, NDVI at heading; PH, Plant height.

Levene’s test for the homogeneity of variances was carried out using the mean values of the phenotypic traits recorded during the two seasons (years) for OE and HE trials. The results of Levene’s test revealed that the variances were equal for all the traits of both OE and HE trials, except NDVI_H in the case of the OE trial (**Supplementary Table S1**). Since the variances were homogeneous across the years, pooled analysis of variance was carried out for the optimum and heat-stressed trials (**Tables 3**, **4**). Under both environments, the genotype effects were significant for all the traits studied, whereas the effects of years and genotype by year were significant for most of the traits. There was notable variation in the temperature and rainfall data among the two cropping seasons (**Table 1**). Due to the extended winter during the 2018-19 season, the BILs displayed delayed flowering and maturity. In the case of the OE2 trial, the mean days to heading increased by 9 days, and the mean days to maturity increased by 11 days in comparison to the OE1 trial (**Table 2**). Similarly, in the heat-stressed trial HE2, the mean days to heading and days to maturity increased by 7 days in comparison to the HE1 trial (**Table 2**).

**Table 3:** Mean square values of the pooled analysis of variance for the optimum environment trial in the *T. durum*-*Ae. speltoides* BILs.

Source	df	PY	TGW	GPS	SN	SL	PL	DTH	DTM	GFD	NDVI_H
Genotype	227	210230.09**	13787.10**	165.61**	6.46**	1.57**	18.91**	36.02**	32.84**	16.66**	0.002**
Replication	1	57.75	2.55	15.47	11.79	1.48	0.63	1.04	8.42	3.53	0.04
Year	1	1904584.50**	47.57**	568.97**	75.05**	0.05	59.24**	17943.44**	32119.67**	2049.05**	0.03**
Genotype X Year	227	3765.14	539.66*	5.39**	1.03**	0.13*	0.65	2.01**	3.67**	1.13*	0.002*

PY, Plot yield; TGW, Thousand-grain weight; GPS, Grains per spike; SN, Spikelet number per spike; SL, Spike length; PL, Peduncle length; DTH, Days to heading; DTM, Days to maturity; GFD, Grain filling duration; NDVI_H, NDVI at heading.

**Significant at p<0.01, *Significant at p<0.05.

**Table 4:** Mean square values of the pooled analysis of variance for the heat-stressed environment trial in the *T. durum*-*Ae. speltoides* BILs.

Source	df	PY	TGW	GPS	SN	SL	PL	DTH	DTM	GFD	NDVI_H
Genotype	227	82762.81**	37.50**	132.10**	3.39**	1.25**	14.23**	17.11**	23.98**	16.64**	0.005**
Replication	1	203118.37	97.67	7.76	3.33	0.05	5.95	9.20	79.83	0.66	0.12
Year	1	1126440.07**	534.20**	566.97**	284.08**	1.38**	115.23**	9344.16**	11333.09**	95.88**	1.51**
Genotype X Year	227	6507.30**	0.85	6.41**	0.70**	0.10*	1.16**	1.42**	1.83**	34.83	0.004**

PY, Plot yield; TGW, Thousand-grain weight; GPS, Grains per spike; SN, Spikelet number per spike; SL, Spike length; PL, Peduncle length; DTH, Days to heading; DTM, Days to maturity; GFD, Grain filling duration; NDVI_H, NDVI at heading.

**Significant at p<0.01, *Significant at p<0.05.

**Figure 3:**
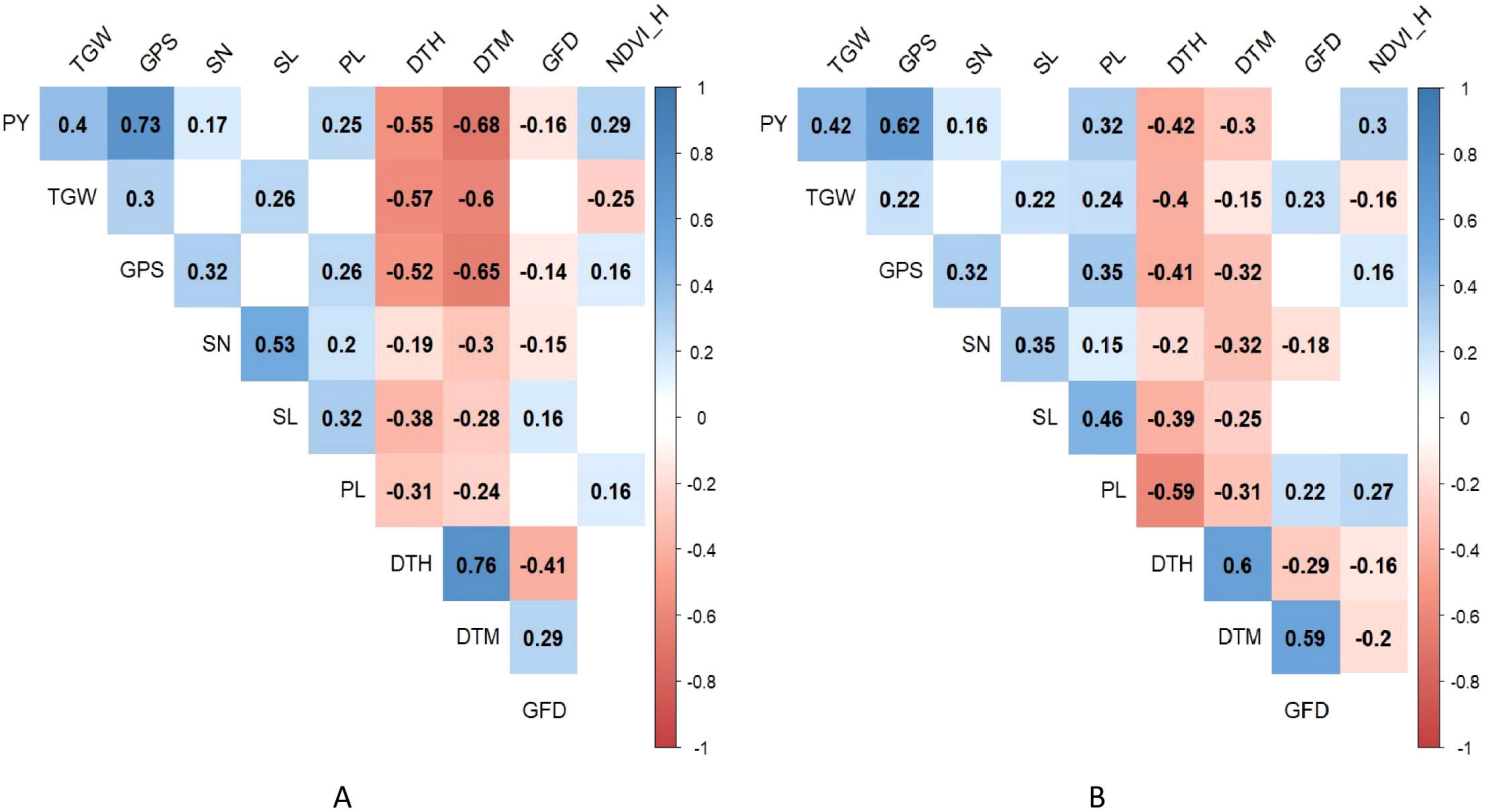
Correlation matrix of the heat tolerance component traits under **(A)** optimum and **(B)** heat-stressed environment.

The degree of correlation among the various heat tolerance component traits under optimum and heat-stressed environments is shown schematically in **Figure 3**. The BLUPs of the phenotypic traits calculated over the two seasons were used to plot the correlation matrix. The plot yield showed a significant positive correlation with TGW, GPS, SN, PL and NDVI_H. A significant negative correlation was observed for plot yield with the DTH and DTM. The GFD displayed a negative and significant correlation with plot yield only under the optimum environment.

### 3.2 Genotyping by sequencing and introgression profiling of the BILs

The distribution of the 2945 high-quality polymorphic SNPs along the 14 chromosomes is given in **Supplementary Figure S6**. The maximum number of polymorphic SNP markers were mapped to chromosome 2B, while chromosome 1A had the least number of SNPs (**Table 5**). Introgression profiling of the BILs indicated multiple introgressions in both the A and B genomes of the durum wheat cv. PDW274 (**Supplementary Figure S7**). Introgressions were observed in all the chromosomes with variable fragment lengths. In some lines, we could observe the entire chromosomes of *Ae. speltoides* being substituted (**Supplementary Figure S7**). The percentage of *Ae*. *speltoides* introgressions in the 14 *T. durum* chromosomes ranged from 4.4 to 17.8%, with an overall mean of 10.5%.

**Table 5:** Statistics of the distribution of the polymorphic SNPs along the 14 chromosomes of the *T. durum*-*Ae. speltoides* BILs.

Chromosome	Number of SNPs	% *T. durum* genome	% *Ae. speltoides* introgressions	% missing data
1A	114	86.4	8.5	5.1
1B	211	86.0	8.6	5.4
2A	217	78.9	16.2	4.9
2B	373	77.9	17.2	4.9
3A	114	83.0	12.5	4.5
3B	190	80.4	13.9	5.7
4A	127	88.2	7.0	4.8
4B	151	89.7	5.2	5.1
5A	258	81.5	13.3	5.2
5B	337	79.0	15.4	5.6
6A	136	88.2	7.1	4.7
6B	318	88.2	6.5	5.3
7A	149	88.8	5.6	5.6
7B	250	89.8	4.7	5.5
Total	2945	84.7	10.1	5.2

### 3.3 QTL mapping for heat stress tolerance component traits

**Figure 4:**
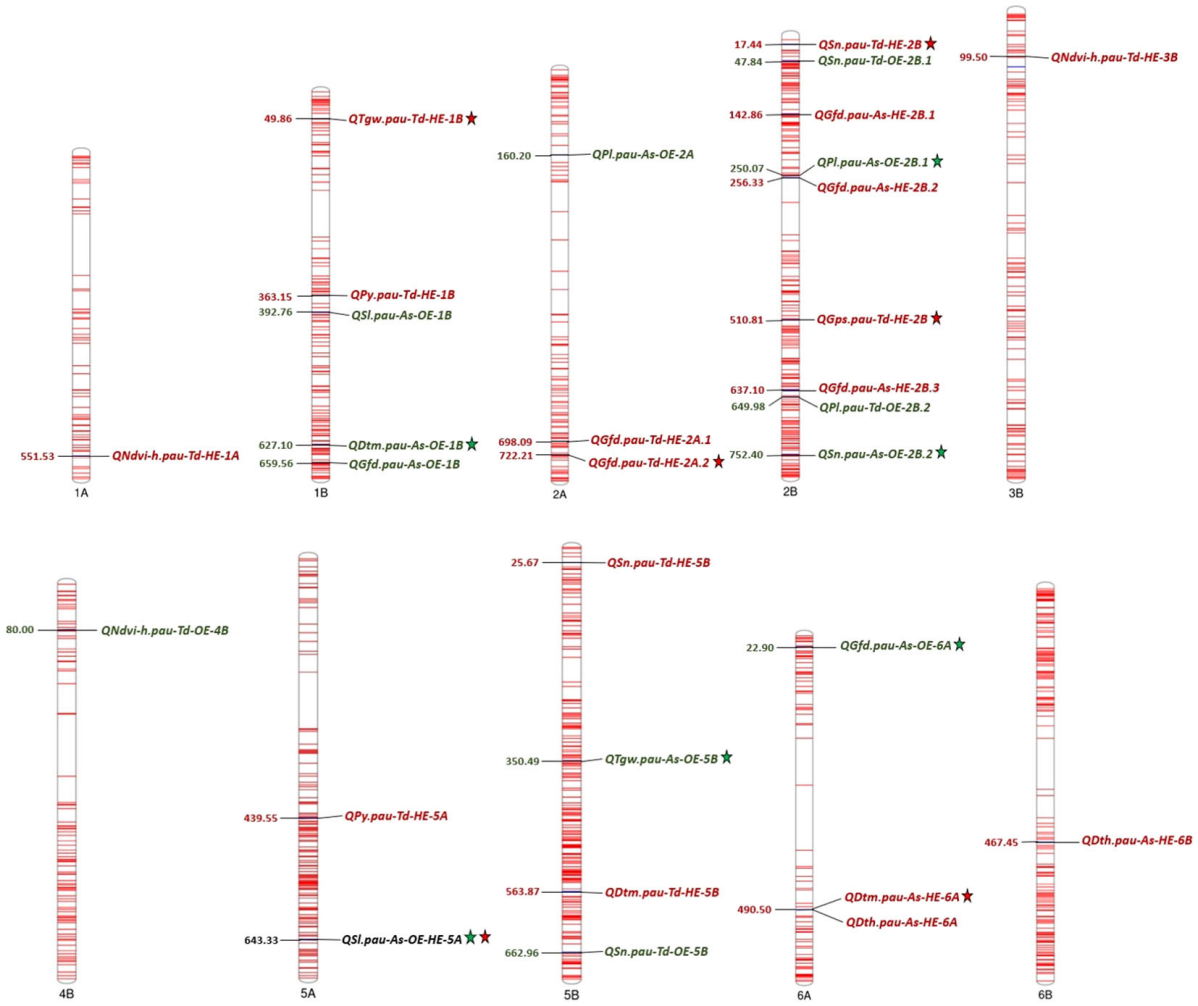
Chromosome map displaying the QTLs detected for various heat tolerance component traits under optimum and heat-stressed environments in the *T. durum*-*Ae. speltoides* BILs. *The numbers on the left side of the chromosomes indicate the physical positions in Mb. Green and red fonts represent QTLs detected in OE and HE. Consistent QTLs are represented by the star symbol.

QTL mapping was carried out using the year-wise and over-the-year BLUPs of all the phenotypic traits except plant height for OE and HE. In total, 30 QTLs were detected under individual and pooled environments. The list of QTLs detected for various heat tolerance component traits is presented in **Table 6**. Out of these, 12 QTLs were detected under OE, 17 QTLs were detected under HE, and one QTL was detected in both OE and HE. The sign of the additive effect denotes the donor of QTLs, like the positive additive effect, which indicates the mapped SNP allele is derived from *Ae. speltoides*, while the negative additive effect represents the contribution by the *T. durum* parent. Concerning QTL density, chromosome 2B had the highest number of mapped QTLs (9 QTLs), and chromosome 1A, 3B, 4B and 6B had the least number (1 QTL each). The mapped QTLs along with their chromosomal positions are visually depicted in **Figure 4**.

**Table 6:** List of QTLs detected for various heat tolerance component traits under the optimum environment (OE) and heat-stressed environment (HE) in the *T. durum*-*Ae. speltoides* BILs.

S. No.	QTL	Trait	SNP marker	Position (Mb)	Environment	LOD	PVE (%)	Additive effect
Optimum environment								
1	*QSn.pau-Td-OE-2B.1*	SN	2B_47840815	47.84	OE_2018-19	5.1	6.9	-0.55
					OE_pooled	3.6	5.0	-0.42
2	*QSn.pau-As-OE-2B.2*	SN	2B_752403466	752.40	OE_2017-18	4.2	6.3	0.36
					OE_2018-19	4.5	6.0	0.38
					OE_pooled	5.3	7.3	0.38
3	*QSn.pau-Td-OE-5B*	SN	5B_662962714	662.96	OE_2017-18	4.3	7.2	-0.57
					OE_pooled	3.4	5.2	-0.49
4	*QSl.pau-As-OE-1B*	SL	1B_392758436	392.76	OE_2017-18	4.5	8.4	0.17
5	*QPl.pau-As-OE-2A*	PL	2A_160196721	160.20	OE_pooled	21.9	6.2	1.59
6	*QPl.pau-As-OE-2B.1*	PL	2B_250066440	250.07	OE_2017-18	2.5	5.2	0.58
					OE_2018-19	12.8	6.6	1.27
					OE_pooled	18.0	5.7	1.52
7	*QPl.pau-Td-OE-2B.2*	PL	2B_649975448	649.97	OE_2018-19	22.2	11.2	-1.82
					OE_pooled	20.2	5.8	-1.71
8	*QNdvi-h.pau-Td-OE-4B*	NDVI_H	4B_80004193	80.00	OE_2018-19	10.7	8.3	-0.01
9	*QTgw.pau-As-OE-5B*	TGW	5B_350487145	350.49	OE_2017-18	3.1	7.5	1.30
					OE_2018-19	2.7	6.3	1.07
					OE_pooled	3.0	7.2	1.21
10	*QGfd.pau-As-OE-1B*	GFD	1B_659555349	659.55	OE_2017-18	4.2	5.4	0.93
11	*QGfd.pau-As-OE-6A*	GFD	6A_22900690	22.90	OE_2017-18	2.5	5.0	0.97
					OE_2018-19	5.0	5.1	1.26
					OE_pooled	3.9	5.3	1.01
12	*QDtm.pau-As-OE-1B*	DTM	1B_627098121	627.10	OE_2017-18	2.9	5.6	1.19
					OE_2018-19	7.8	7.5	1.86
					OE_pooled	11.4	8.4	2.27
Heat stressed environment								
13	*QSn.pau-Td-HE-2B*	SN	2B_17444376	17.44	HE_2017-18	2.5	5.1	-0.21
					HE_2018-19	3.0	5.1	-0.23
					HE_pooled	2.9	5.0	-0.24
14	*QSn.pau-Td-HE-5B*	SN	5B_25666540	25.67	HE_2017-18	3.3	6.2	-0.56
					HE_pooled	3.8	6.6	-0.50
15	*QPy.pau-Td-HE-1B*	PY	1B_363149235	363.15	HE_2018-19	7.9	8.7	-89.06
					HE_pooled	7.6	8.6	-88.84
16	*QPy.pau-Td-HE-5A*	PY	5A_439551042	439.55	HE_2017-18	2.9	6.1	-52.4
17	*QNdvi-h.pau-Td-HE-1A*	NDVI_H	1A_551533041	551.53	HE_2017-18	7.8	9.2	-0.01
18	*QNdvi-h.pau-Td-HE-3B*	NDVI_H	3B_99501367	99.50	HE_2018-19	9.3	6.7	-0.01
19	*QTgw.pau-Td-HE-1B*	TGW	1B_49863151	49.86	HE_2017-18	3.2	5.4	-1.46
					HE_2018-19	2.7	5.3	-1.27
					HE_pooled	2.8	5.5	-1.34
20	*QGps.pau-Td-HE-2B*	GPS	2B_510812119	510.81	HE_2017-18	3.2	6.1	-2.18
					HE_2018-19	2.5	5.0	-1.90
					HE_pooled	2.6	5.1	-1.97
21	*QGfd.pau-Td-HE-2A.1*	GFD	2A_698093562	698.09	HE_2017-18	8.1	6.2	-0.72
22	*QGfd.pau-Td-HE-2A.2*	GFD	2A_722208150	722.21	HE_2017-18	6.1	5.0	-0.67
					HE_2018-19	12.5	8.6	-1.11
					HE_pooled	8.5	5.3	-0.76
23	*QGfd.pau-As-HE-2B.1*	GFD	2B_142863752	142.86	HE_2018-19	13.3	8.3	1.12
24	*QGfd.pau-As-HE-2B.2*	GFD	2B_256328903	256.33	HE_2017-18	14.7	10.6	0.96
					HE_pooled	16.9	10.8	1.10
25	*QGfd.pau-Td-HE-2B.3*	GFD	2B_637100920	637.10	HE_pooled	9.4	6.0	-0.80
26	*QDtm.pau-Td-HE-5B*	DTM	5B_563875919	563.87	HE_2017-18	4.1	5.8	-0.96
					HE_pooled	3.4	5.0	-0.87
27	*QDtm.pau-As-HE-6A*	DTM	6A_490496402	490.50	HE_2017-18	4.4	6.4	0.78
					HE_2018-19	3.8	5.0	0.72
					HE_pooled	4.3	6.1	0.74
28	*QDth.pau-As-HE-6A*	DTH	6A_490496402	490.50	HE_2017-18	2.9	7.2	0.54
29	*QDth.pau-As-HE-6B*	DTH	6B_467448196	467.45	HE_2018-19	9.0	6.3	2.11
					HE_pooled	9.7	6.7	2.04
Optimum and Heat stressed environment								
30	*QSl.pau-As-OE-HE-5A*	SL	5A_643325049	643.32	OE_2018-19	3.6	8.4	0.27
					OE_pooled	3.6	8.3	0.25
					HE_2017-18	3.4	6.3	0.22
					HE_2018-19	4.9	9.6	0.26
					HE_pooled	4.4	8.5	0.24

PY, Plot yield; TGW, Thousand-grain weight; GPS, Grains per spike; SN, Spikelet number per spike; SL, Spike length; PL, Peduncle length; DTH, Days to heading; DTM, Days to maturity; GFD, Grain filling duration; NDVI_H, NDVI at heading.

With respect to the OE, 12 QTLs were detected for SN, SL, PL, NDVI_H, TGW, GFD and DTM on chromosome 1B, 2A, 2B, 4B, 5B and 6A respectively (**Table 6**). Of these, 5 QTLs, *QSn.pau-As-OE-2B.2, QPl.pau-As-OE-2B.1*, *QTgw.pau-As-OE-5B, QGfd.pau-As-OE-6A* and *QDtm.pau-As-OE-1B* were consistently detected across the years (**Table 6**). The phenotypic variance explained (PVE) ranged from 5 to 11.2%. In the case of HE, 17 QTLs were detected for the SN, PY, NDVI_H, TGW, GPS, GFD, DTH and DTM on chromosomes 1A, 1B, 2A, 2B, 3B, 5A, 5B, 6A and 6B. QTLs that were detected across both the years include *QSn.pau-Td-HE-2B*, *QTgw.pau-Td-HE-1B*, *QGps.pau-Td-HE-2B*, *QGfd.pau-Td-HE-2A.2* and *QDtm.pau-As-HE-6A* (**Table 6**). The PVE of the detected QTLs ranged from 5 to 10.8%. The QTL for SL *QSl.pau-As-OE-HE-5A* on chromosome 5A was detected under both OE and HE, with the PVE ranging from 6.3 to 9.6%.

### 3.4 Testing the allelic effect of the SNP associated with the stable QTLs

**Figure 5:**
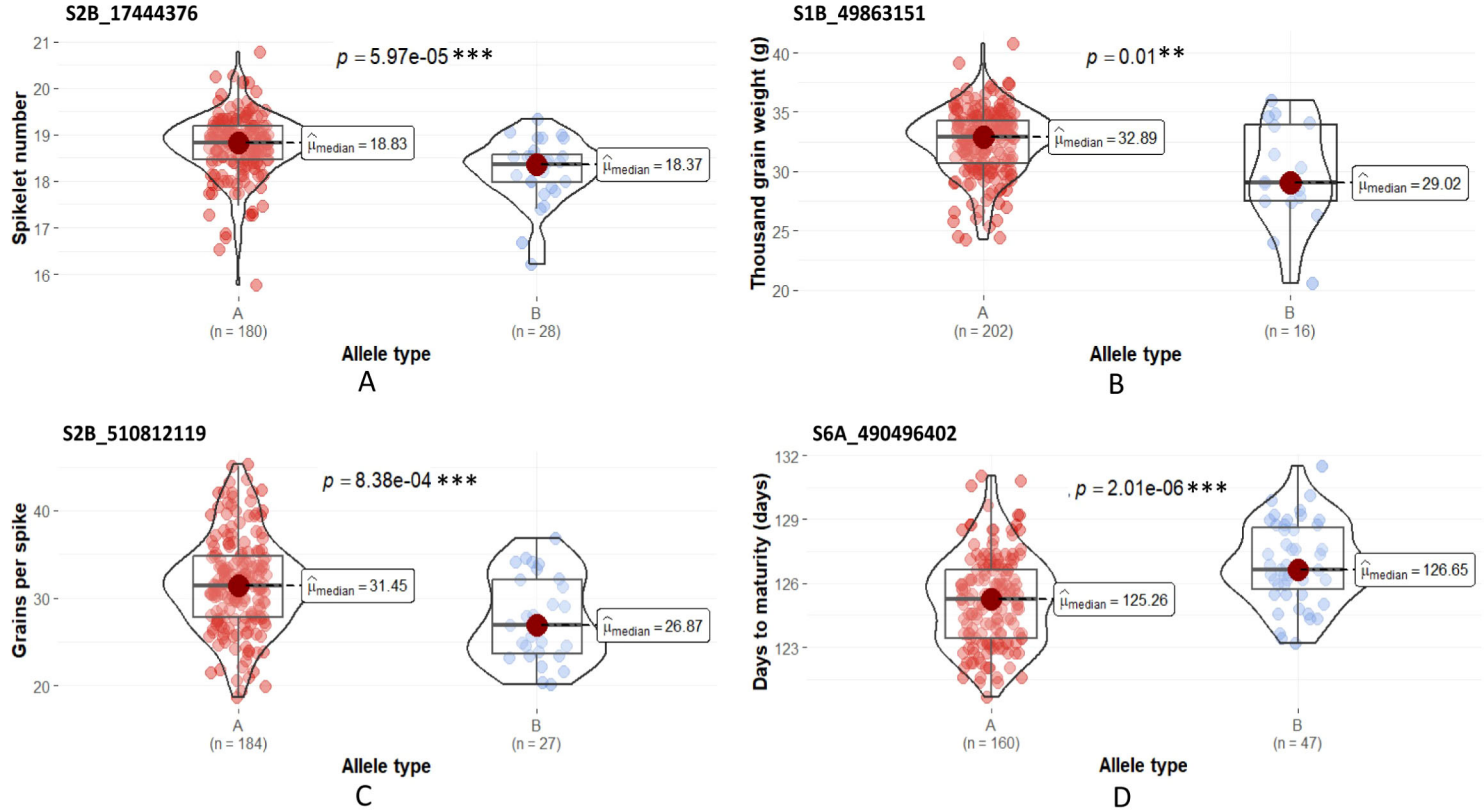
Allelic effects of alternative SNPs at the QTL locus. **(A)** *QSn.pau-Td-HE-2B* (S2B_17444376), **(B)** *QTgw.pau-Td-HE-1B* (S1B_49863151), **(C)** *QGps.pau-Td-HE-2B* (S2B_510812119) and **(D)** *QDtm.pau-As-HE-6A* (S6A_490496402). ‘A’ refers to the allele type of the durum parent and ‘B’ refers to the allele type of Ae. speltoides. ***Significant at α = 0.01, **Significant at α = 0.05.

The effect contributed by the alleles linked to the stable QTLs was studied by comparing the phenotypic distributions of the genotype groups sorted based on the allelic pattern at the SNP locus. The stable QTLs detected under the HE were used for the analysis. The Mann-Whitney U test revealed significant differences in the median phenotypic performance among the alternative allelic groups for four out of the five stable QTLs tested (**Figure 5**). The difference in the median phenotypic performance of the allelic group was insignificant in the case of the QTL for GFD *QGfd.pau-Td-HE-2A.2*. The SNP allele linked to all these stable QTLs, excluding *QDtm.pau-As-HE-6A*, was contributed by *T. durum* parent.

### 3.5 Postulation of putative candidate genes in the significant QTL regions mapped under the heat-stressed environment

The genome-wide LD decay value was estimated to be 1.85 Mb (**Supplementary Figure S8**). The 1.85 Mb physical region flanking the SNPs linked to the consistent QTLs detected under the HE revealed the presence of multiple genes known to modulate heat stress responses. The annotations of the postulated candidate genes are given in **Table 7**. A total of 21 genes were selected after scanning the 1.85 Mb interval flanking the five consistent QTLs based on the gene annotation and literature search. The proteins encoded by these putative candidate genes include cytochrome P450, lectin receptor kinase, E3 ubiquitin-protein ligase, arginine/serine-rich splicing factor, ABC transporter protein, peptidyl-prolyl cis-trans isomerase, alcohol dehydrogenase, F-box protein, plastid-lipid associated protein and MYB family protein.

**Table 7:** List of postulated candidate genes for the consistent QTLs detected under the heat-stressed environment in the *T. durum-Ae. speltoides* BILs.

S. No.	QTL	Trait	Chromo-some	Mapped SNP position (Mb)	Distance from SNP (Kb)	Gene stable ID	Function
1	*QSn.pau-Td-HE-2B*	SN	2B	17.44	-333.773	*TraesCS2B01G035600*	Cytochrome P450
					-300.309	*TraesCS2B01G035700*	Cytochrome P450
					+55.28	*TraesCS2B01G036600*	Lectin receptor kinase
					-4.197	TraesCS2B01G036900	Cytochrome P450
					-0.51	*TraesCS2B01G037000*	Cytochrome P450
					+58.886	*TraesCS2B01G037600*	Cytochrome P450
					+70.952	*TraesCS2B01G037700*	Cytochrome P450
2	*QTgw.pau-Td-HE-1B*	TGW	1B	49.86	-643.722	*TraesCS1B01G064800*	Cytochrome P450
					-69.26	*TraesCS1B01G065800*	E3 ubiquitin-protein ligase RNF14
					-320.63	*TraesCS1B01G065900*	Arginine/serine-rich splicing factor
					-340.66	*TraesCS1B01G066000*	ABC transporter G family member
					+537.015	*TraesCS1B01G066300*	Peptidyl-prolyl cis-trans isomerase
					+911.743	*TraesCS1B01G066400*	Alcohol dehydrogenase
3	*QGps.pau-Td-HE-2B*	GPS	2B	510.81	-754.56	*TraesCS2B01G357000*	F-box family protein
					-320.48	*TraesCS2B01G358200*	ABC transporter ATP-binding protein
					+418.56	*TraesCS2B01G357200*	Cytochrome P450
4	*QGfd.pau-Td-HE-2A.2*	GFD	2A	722.21	+0.096	*TraesCS2A01G487600*	F-box protein
					-311.44	*TraesCS2A01G487900*	Plastid-lipid associated protein/fibrillin family protein
					+516.360	*TraesCS2A01G488200*	MYB-like transcription factor family protein
					+894.275	*TraesCS2A01G489200*	F-box protein
5	*QDtm.pau-As-HE-6A*	DTM	6A	490.49	+194.61	*TraesCS6A01G265300*	MYB family protein

(Positive sign of the distance from SNP denotes that the gene was present upstream of the mapped QTL (SNP) position and negative sign indicates that the gene was present downstream of the mapped QTL (SNP) position).

TGW, Thousand-grain weight; GPS, Grains per spike; SN, Spikelet number per spike; DTM, Days to maturity; GFD, Grain filling duration.

## 4 Discussion

### 4.1 Phenotypic evaluation of BILs for heat stress tolerance and introgression profiling

The BILs displayed significant variation for all the heat tolerance component traits studied. Due to the unseasonal rainfall during the 2018-19 trial, the HE2 trial recorded a decline in the plot yield and NDVI. This was due to the reduction in viability and growth of the BILs sown in the HE2 trial. Correlation analysis indicated that the early flowering and early maturing genotypes produce better yields during terminal heat stress possibly due to escape from high temperature stress. Selection for higher TGW, GPS, SN, PL and NDVI_H can help in achieving better yields in both environments.

Introgression profiling of the BILs revealed random introgressions of donor chromosome fragments across all the 14 chromosomes of the recurrent parent. This phenomenon was observed likely due to the *Ph1* suppression activity of the *Ae. speltoides* accession pau3809, which would have induced the crossing over between the S genome of *Ae. speltoides* and both the A and B genomes of the durum wheat cv. PDW274 (Dvorak et al., 2006; Millet, 2008).

### 4.2 QTL mapping

The QTLs were mapped under OE and HE for the two cropping seasons. Out of the 12 QTLs detected under OE, five QTLs were detected consistently over the two seasons. The consistent QTL for SN *QSn.pau-As-OE-2B.2*, contributed by *Ae. speltoides*, was mapped on chromosome 2B. Reports on the mapping of SN QTLs on chromosome 2B are available in the studies of Edae et al. (2014) and Li et al. (2021). Stable QTL for PL *QPl.pau-As-OE-2B.1* was mapped on chromosome 2B, as reported earlier by Neumann et al., 2011 and Graziani et al., 2014. A stable QTL for TGW *QTgw.pau-As-OE-5B* was located on chromosome 5B at a physical distance of 350.49 Mb, with a maximum PVE of 7.5%. A major and stable QTL for TGW *QTgw.caas-5B* was fine-mapped in the 5B chromosome within the physical interval of 49.6 Mb-51.6 Mb in a RIL population derived from Zhongmai871 and Zhongmai895 (Zhao et al., 2021). Results on mapping TGW QTLs on the same chromosome were obtained by Ramya et al. (2010); Zhang et al. (2018); Dhakal et al. (2021); Amalova et al. (2021) and Ahmed et al. (2022). The stable QTL controlling grain filling duration was detected on the 6A chromosome and localized to a physical interval of 22.90 Mb. To the best of our knowledge, this is the first report of mapping a GFD QTL on the 6A chromosome. A meta-QTL MQTL6A.3 for multiple traits, including grain number, spike-related traits, grain morphology-related traits and days to heading, was found to span this QTL and was delimited within the physical interval of 12.4 Mb to 43.4 Mb (Saini et al., 2022). The stable QTL for DTM *QDtm.pau-As-OE-1B* was detected on chromosome 1B, with the PVE ranging from 5.6 to 8.4%. Similar results are available in the studies of Cuthbert et al. (2008) and Kamran et al. (2013).

In the case of HE, 17 QTLs were detected in total, and among those, five QTLs were detected across the seasons. Consistent QTLs were detected for the traits SN, TGW, GPS, GFD and DTM. The stable QTL for SN *QSn.pau-Td-HE-2B* was detected on chromosome 2B. Earlier workers had reported mapping of SN QTLs on chromosome 2B (Qaseem et al., 2019; Telfer et al., 2021). The stable TGW QTL *QTgw.pau-Gw-Td-HE-1B* was detected on chromosome 1B at a physical distance of 49.86 Mb with a maximum PVE of 5.5%. Pankaj et al. (2024) identified a stable QTL for TGW located close to the mapped QTL and flanked by the markers *Xwmc44* and *Xwmc367* on chromosome 1B. Tahmasebi et al. (2016) and Ogbonnaya et al. (2017) also detected TGW QTLs on the same chromosome. The consistent QTL for GPS *QGps.pau-Td-HE-2B* was mapped to the 2B chromosome, with the PVE ranging from 5 to 6.1%. Previous reports are available on mapping of GPS QTLs on chromosome 2B (Tahmasebi et al., 2016; Sharma et al., 2016; Li et al., 2019; Elbashier et al., 2023). The consistent QTL for GFD *QGfd.pau-Td-HE-2A.2* was located on chromosome 2A at a physical distance of 722.21 Mb with a maximum PVE of 12.5%. Reports on the mapping of GFD QTLs on chromosome 2A are found in the publications of Mason et al. (2010) and Pankaj et al. (2024). The photoperiod sensitivity gene *Ppd-A1*, responsible for the initiation of flowering in wheat, was reported to be located on the short arm of chromosome 2A, and mutations in this gene enable the plants to flower early (Law et al., 1978; Wilhelm et al., 2009; Achilli et al., 2022). Early flowering spring wheat genotypes tend to have longer grain filling durations and produce better yields, especially under terminal heat stress (Vinod et al., 2012). For the trait DTM, a stable QTL was detected on chromosome 6A and a similar result was documented by Qaseem et al. (2019).

A QTL for SL *QSl.pau-As-OE-HE-5A* was detected consistently across the years under both OE and HE. Many reports are available on mapping of SL QTL on chromosome 5A under non-stress conditions (Kumar et al., 2007; Wang et al., 2011; Ma et al., 2014; Kang et al., 2020). A stable QTL for SL was localized to a 6.69 Mb interval (518.4-525.1 Mb) on chromosome 5A, explaining 7.8 to 26.6% PVE (Ji et al., 2021). Under heat-stressed conditions, no published studies are available on detecting SL QTLs on this chromosome, suggesting it may be a novel QTL.

### 4.3 Allelic effect of the SNP associated with the stable QTLs on the linked phenotypic trait

The effect of the SNP allele associated with the stable QTLs mapped under HE on the phenotypic performance of the BILs was compared with the alternative allele using Mann-Whitney U test. The allelic effects were significant for four out of the five stable QTLs. These results validate that the QTL alleles significantly contributed to the enhancement of the associated phenotypic trait.

### 4.4 Postulation of putative candidate genes in the significant QTL regions mapped under the heat-stressed environment and their role in heat stress

Several genes were found in the 1.85 Mb physical interval flanking the five consistent QTLs detected under the HE. The gene annotations were used to perform a literature search to identify their role in heat stress regulation. Accordingly, 21 putative candidate genes were shortlisted and their role in mediating heat stress response will be discussed in the following sections.

Seven genes were found in the physical interval flanking the QTL for SN *QSn.pau-Td-HE-2B*, encoding cytochrome P450 and lectin receptor kinase. Shumayla et al. (2016) conducted a global expression analysis of bread wheat lectin receptor kinases (LRK) genes and identified 263 LRKs, out of which 77 LRKs were differentially expressed under heat, drought or a combination of both stresses. About 40% of the rice Lectin RLKs identified from the genome-wide analysis were differentially expressed during heat stress treatment (Vaid et al., 2012). Eighteen Lectin RLK genes were upregulated in foxtail millet plants treated with 6% polyethylene glycol (PEG-6000), simulating drought and high temperature stress conditions (Zhao et al., 2016). Multiple transcripts of Cytochrome P450 (CYP) were up- or down-regulated during the heat stress treatment at 40 °C for 10 hour in perennial ryegrass and tall fescue (Tao et al., 2017). Heat stress induced the expression of 11 different CYP genes in switchgrass, and two genes belonging to the CYP71A1 family involved in the secretion of indole alkaloid secologanin were upregulated (Li et al., 2013a). The hormone abscisic acid (ABA) plays a role in the arrest of plant growth during biotic or abiotic stresses and growth phase transitions in plant development (Umezawa et al., 2010). Genes responsible for ABA catabolism (*CYP707A* family genes) were downregulated during heat stress in Arabidopsis (Baron et al., 2012).

The region flanking the stable QTL *QTgw.pau-Td-HE-1B* was found to contain 6 genes coding for cytochrome P450, ubiquitin-protein ligase RNF14, arginine/serine-rich splicing factor, ABC transporter G family member, peptidyl-prolyl cis-trans isomerase and alcohol dehydrogenase. The rice *OsDHSRP1* gene, a RING finger E3 ligase, negatively regulates heat, drought and salt stress responses by degrading the glyoxalase protein through the Ub/26S proteasome system (Kim et al., 2020). Liu et al. (2020) reported the involvement of Arabidopsis *AtPPRT1* gene, a zinc finger ubiquitin E3 ligase, in conferring basal and acquired thermotolerance by mediating the degradation of uncharacterized proteins. T-DNA insertion mutants for the *AtPPRT1* gene displayed lower germination and survival rates when exposed to high-temperature stress. Palusa and Reddy (2015) elucidated the involvement of spliced isoforms of serine- and arginine-rich (SR) proteins during heat stress in Arabidopsis. Heat priming in Arabidopsis induced the expression of alternatively spliced heat shock factors (HSFs) and SR transcripts *SR30*, *SR45a*, *SR34*, and *RS41*, implicating the role of these proteins in stress memory (Ling et al., 2018). Exposure of Arabidopsis plants to combined drought and heat stress upregulated the expression levels of the ATP-binding cassette (ABC) transporter gene *At1g64550* (Rizhsky et al., 2004). Gupta et al. (2019) characterized the plant-specific ABC receptors termed ABCG receptors in rice and identified multiple ABC receptor genes showing stress-specific expression patterns under various abiotic stresses like drought, heat and salinity. Wang et al. (2016) imposed heat priming on wheat plants at seedling and flowering stages and observed an upregulation of the *cyclophilin 38* gene, a type of peptidyl-prolyl cis-trans isomerase (PPI) when the progeny of primed plants were exposed to post anthesis heat stress. Genome-wide analysis of cyclophilin genes in wheat led to the identification of 83 cyclophilin genes distributed in all 21 wheat chromosomes. Some of these genes housed a heat shock element in their regulatory region and were known to modulate heat stress responses (Singh et al., 2019). Li et al. (2013b) carried out a proteomic analysis of the leaf tissues of alfalfa plants exposed to different periods of heat stress. The alcohol dehydrogenase (ALDH) protein displayed a four-fold higher expression under a 48-hour heat stress treatment compared to the control plants. The Arabidopsis ALDH genes *ALDH3I1* and *ALDH7B4*, known to participate in various abiotic stress responses, were strongly induced by high-temperature stress, and the double knockout mutants displayed severe heat sensitivity (Zhao et al., 2017a).

The QTL for GPS *QGps.pau-Td-HE-2B* houses three genes encoding the F-box family protein, ABC transporter ATP-binding protein and cytochrome P450. The functions of ABC transporter protein and cytochrome P450 are already discussed in the preceding sections. Stefanowicz et al. (2015) elaborated the role of plant F-box proteins as regulatory players of proteolytic mechanisms in response to cellular stimuli during hormone signalling, abiotic and biotic stresses and plant morphogenesis. Li et al. (2018) overexpressed the wheat F-box gene *TaFBA1* in transgenic tobacco plants, followed by exposure of the transgenics to heat stress. The transgenic plants exhibited lesser growth inhibition and increased photosynthesis compared to the control. Genes involved in ROS scavenging, proline biosynthesis and abiotic stress regulation were upregulated in the transgenic plants.

The grain filling duration QTL *QGfd.pau-Td-HE-2A.2* encompasses four genes coding for F-box protein, plastid-lipid associated protein/fibrillin family protein and MYB-like transcription factor family protein, respectively, within the 1.85 Mb interval. Genome-wide analysis and expression characterization of rice fibrillin genes (*OsFBNs*), led to the identification of 11 fibrillin genes (*OsFBN1* to *OsFBN11*) that were upregulated during heat stress (Li et al., 2020). Cis-acting elements involved in hormonal signalling, photoreaction and environmental stresses were detected upstream to these genes. Jiang et al. (2020) identified and characterized 26 FBN genes distributed on 11 wheat chromosomes using genome-wide analysis and expression studies. The wheat FBN genes *TaFBN-A1, TaFBN-B1, TaFBN-A2, TaFBN-B2, TaFBN-D2, TaFBN-D6* and *TaFBN-B6* were significantly upregulated during abiotic and biotic stress treatments, including heat, implicating their role in stress responses. MYB30, an R2R3 type MYB transcription factor, regulates oxidative and heat stress responses in Arabidopsis by controlling cytosolic calcium signalling (Liao et al., 2017). The mutant plants for the *MYB30* gene were sensitive to heat stress. Six heat-induced MYB genes (*TaMYB*s) were identified from transcriptome data derived from wheat plants subjected to heat stress (Zhao et al., 2017b). One of the MYB genes, *TaMYB80*, conferred heat and drought tolerance when expressed in Arabidopsis on account of increased cellular levels of ascorbic acid. Jacob et al. (2021) identified two MYB transcription factor genes, *TT2* and *MYB5*, that are co-regulated along with Heat Shock Factor A2, which is a regulator of multiple environmental stresses in Arabidopsis. Overexpression of the *TT2* and *MYB5* genes conferred enhanced heat stress tolerance in the transgenic plants, whereas the *tt2*, *myb5* and *tt2/myb5* loss of function mutants displayed heat sensitivity. A gene encoding MYB family protein was located in the 1.85 Mb interval flanking the stable QTL for DTM *QDtm.pau-As-HE-6A*. The role of this gene was discussed in the previous sections.

## 5 Conclusion

Negative effects of heat stress on wheat yields has caused serious concerns for wheat growers in recent times. For wheat production to be sustained in the near future, it is essential to identify genetic regions contributing to heat tolerance in the exotic and wild wheat germplasm. In an attempt to uncover genomic regions controlling heat tolerance-related traits, we conducted a QTL mapping study on a set of *T. durum-Ae. speltoides* BILs. Thirty QTLs were detected for various heat tolerance component traits under optimum and heat-stressed environments in multiple wheat chromosomes. Stable QTLs were identified for some of the traits under heat-stressed environment for use in marker-assisted selection (MAS). The 1.85 Mb physical interval flanking the stable heat tolerance QTLs harboured genes known to play essential roles in regulating heat stress responses. The BILs carrying multiple heat tolerance QTL can be employed in wheat breeding programmes for improving heat tolerance using MAS.

## Data Availability

The original contributions presented in the study are included in the article/**Supplementary Material**. Further inquiries can be directed to the corresponding author.
