# Supplementary material for: Mapping heat tolerance QTLs in Triticum durum-Aegilops speltoides backcross introgression lines to enhance thermotolerance in wheat
Source: Front Plant Sci. 2024 Dec 20;15:1485914. doi: 10.3389/fpls.2024.1485914 (PMC11695302; doi:10.3389/fpls.2024.1485914)
Supplement: Supplementary file 1 [file DataSheet1.pdf]

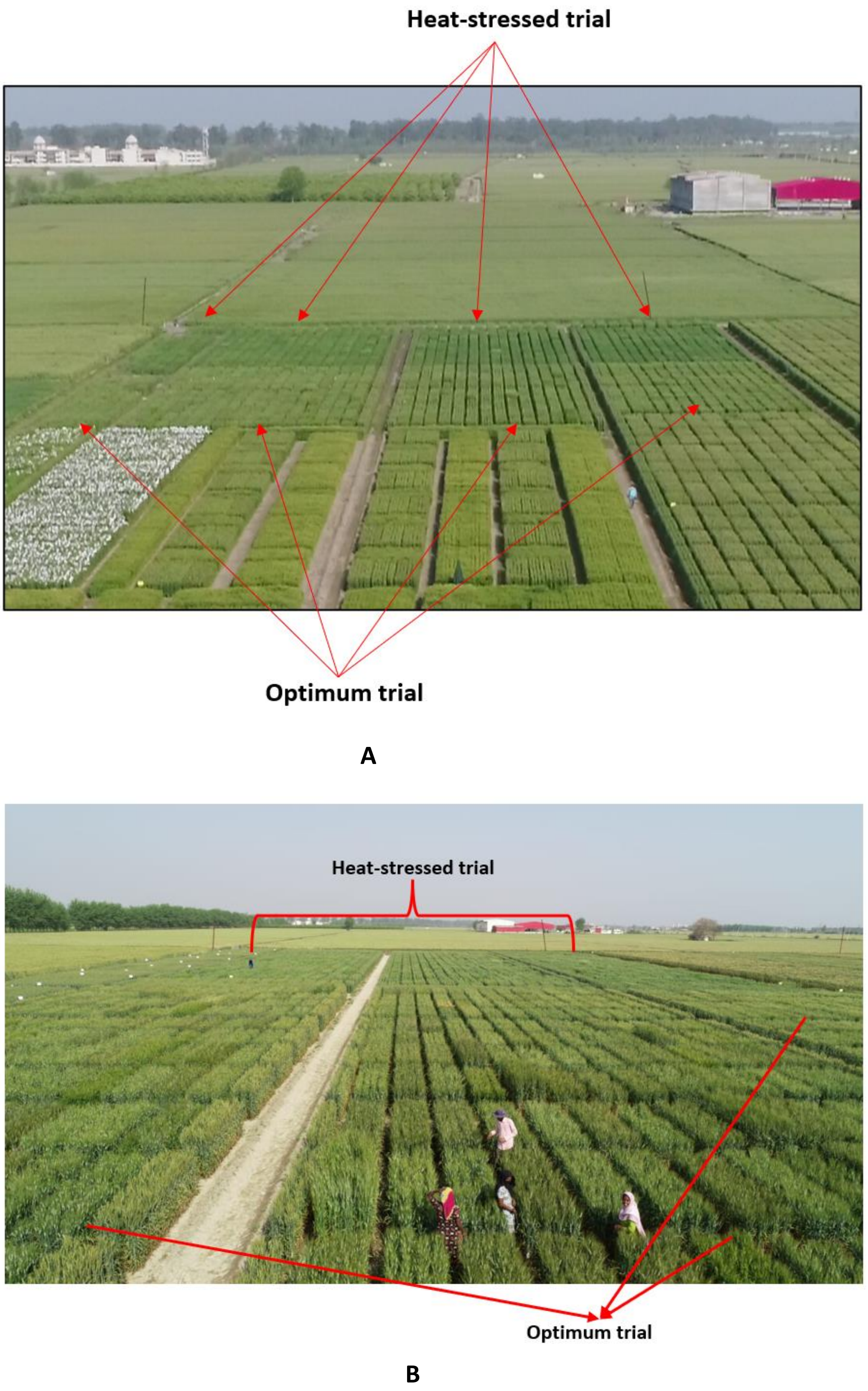

Supplementary Figure S1 Aerial view of the optimum and heat-stressed field trials. (A) 2017-18 season and (B) 2018-19 season.

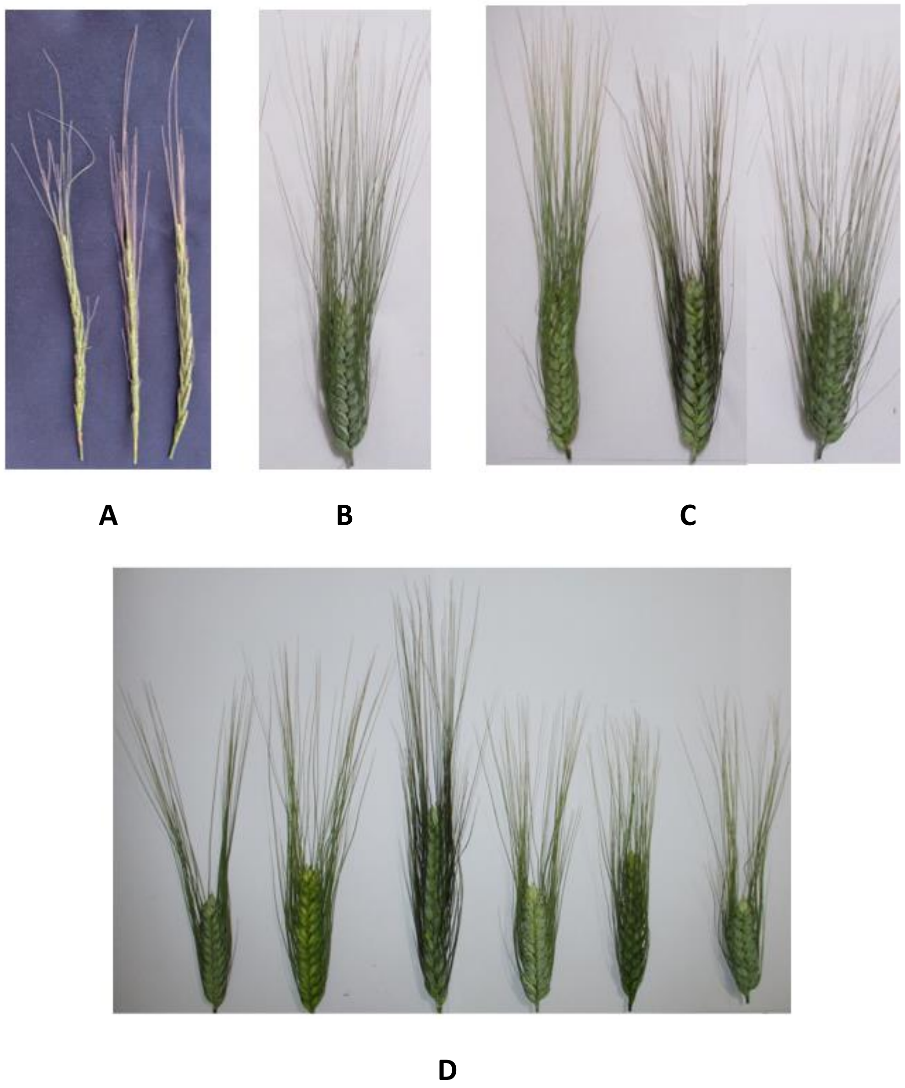

Supplementary Figure S2 Variations in spike morphology among the *T. durum*-*Ae. speltoides* BILs: (A) *Ae. speltoides* acc. pau3809, (B) *T. durum* cv PDW274, (C) and (D) BILs.

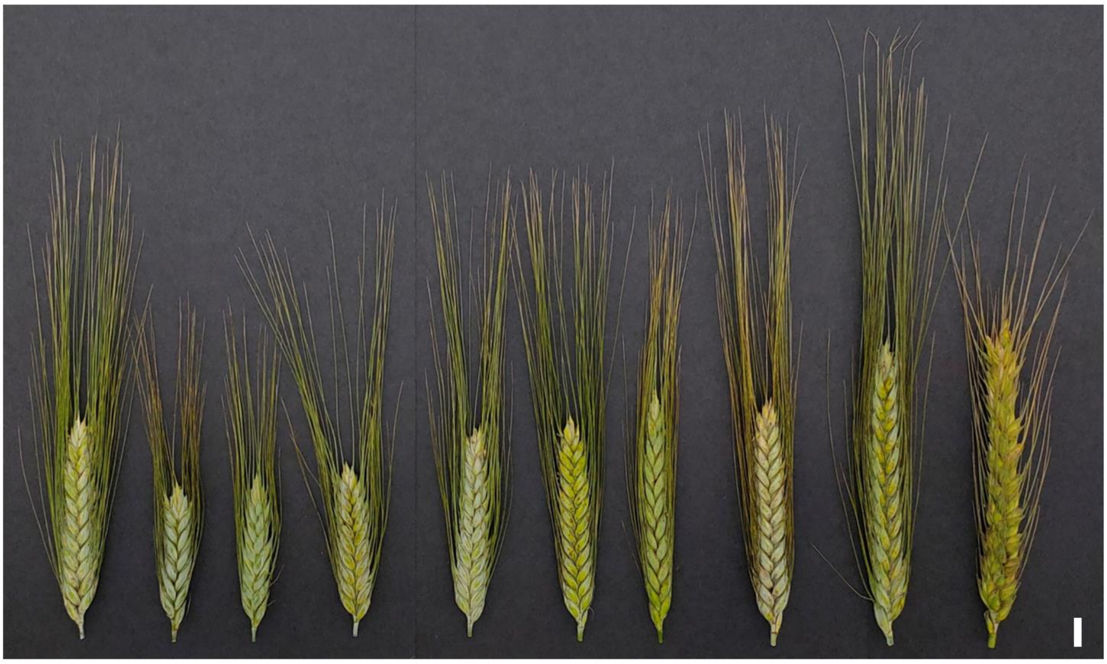

PDW274 DS326 DS59 DS73 DS49 DS38 DS69 DS54 DS439 DS394

A

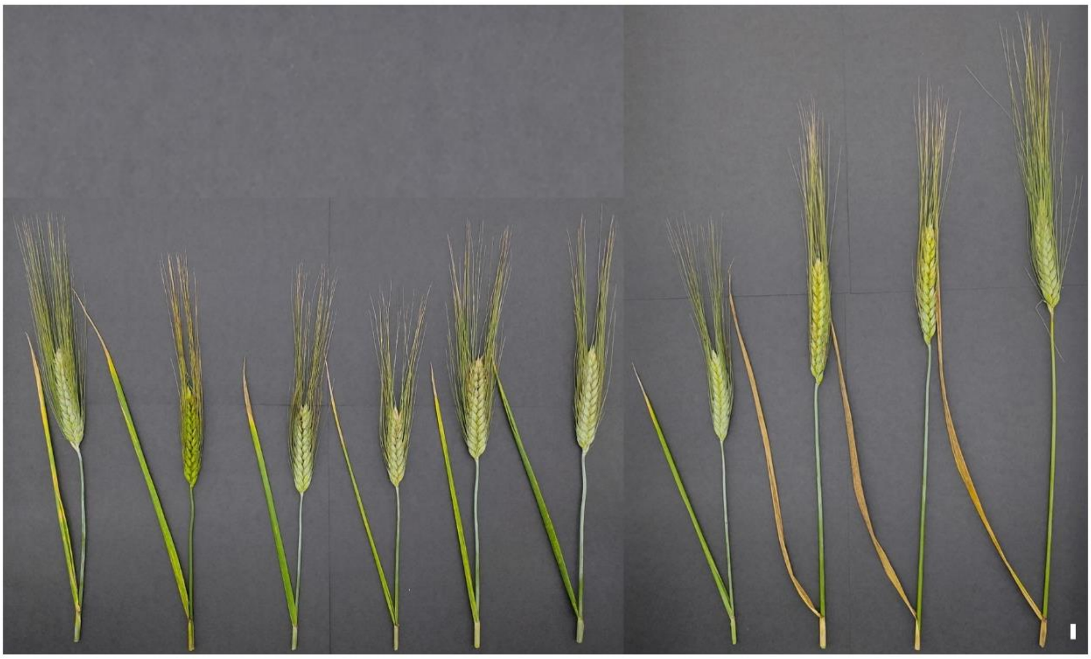

PDW274 DS172 DS301 DS371 DS100 DS240 DS204 DS350 DS389 DS385

B

Supplementary Figure S3 Variation for (A) Spike length and (B) Peduncle length in the BILs and the recurrent parent PDW274. 1 scale bar = 1cm.

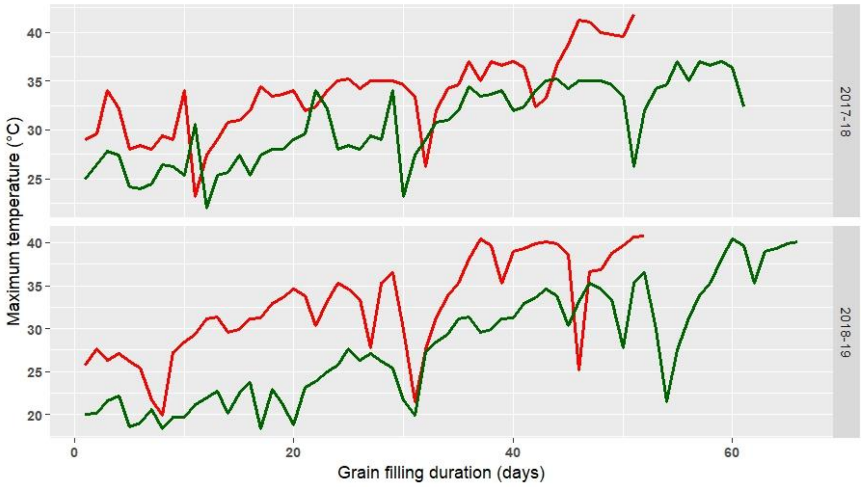

A

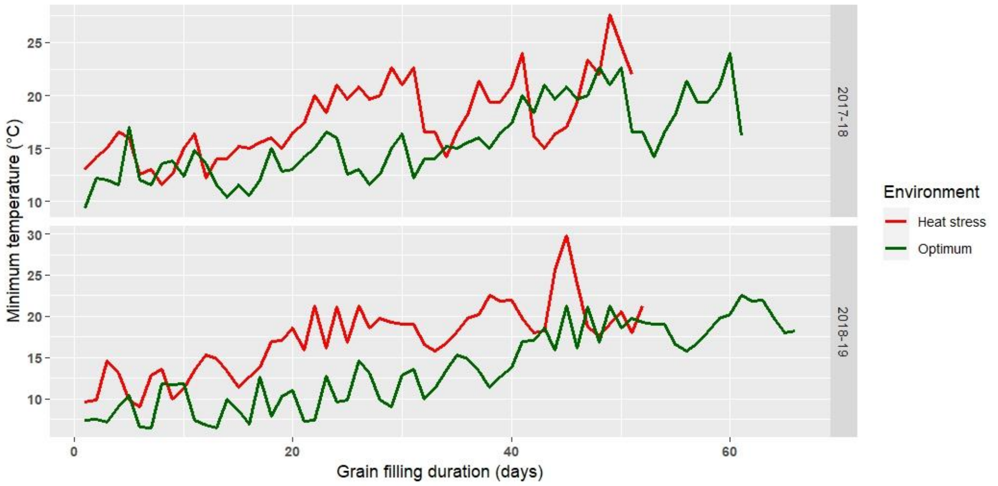

B

Supplementary Figure S4 Variations in (A) daily maximum and (B) daily minimum temperatures during the grain filling period for the two cropping seasons 2017-18 and 2018-19

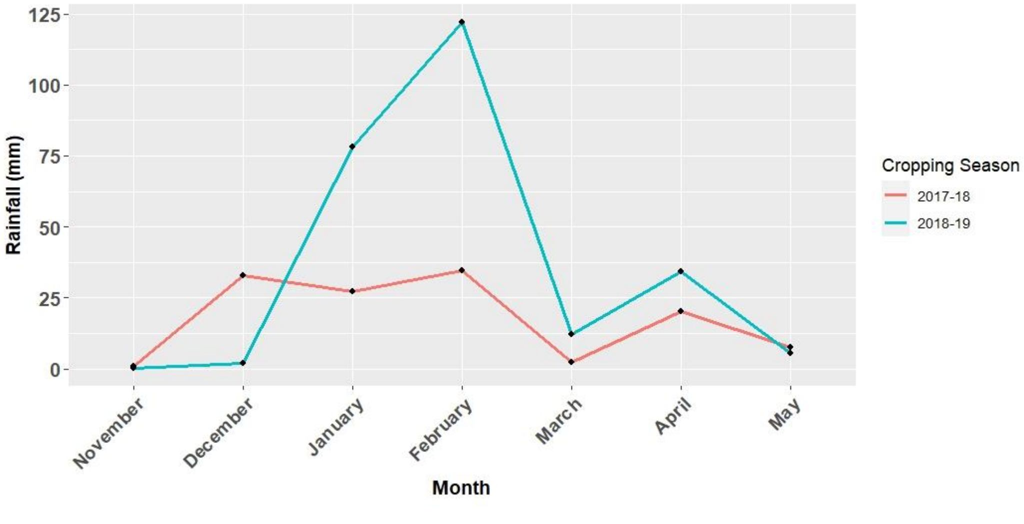

Supplementary Figure S5 Monthly rainfall received during the two cropping seasons 2017-18 and 2018-19.

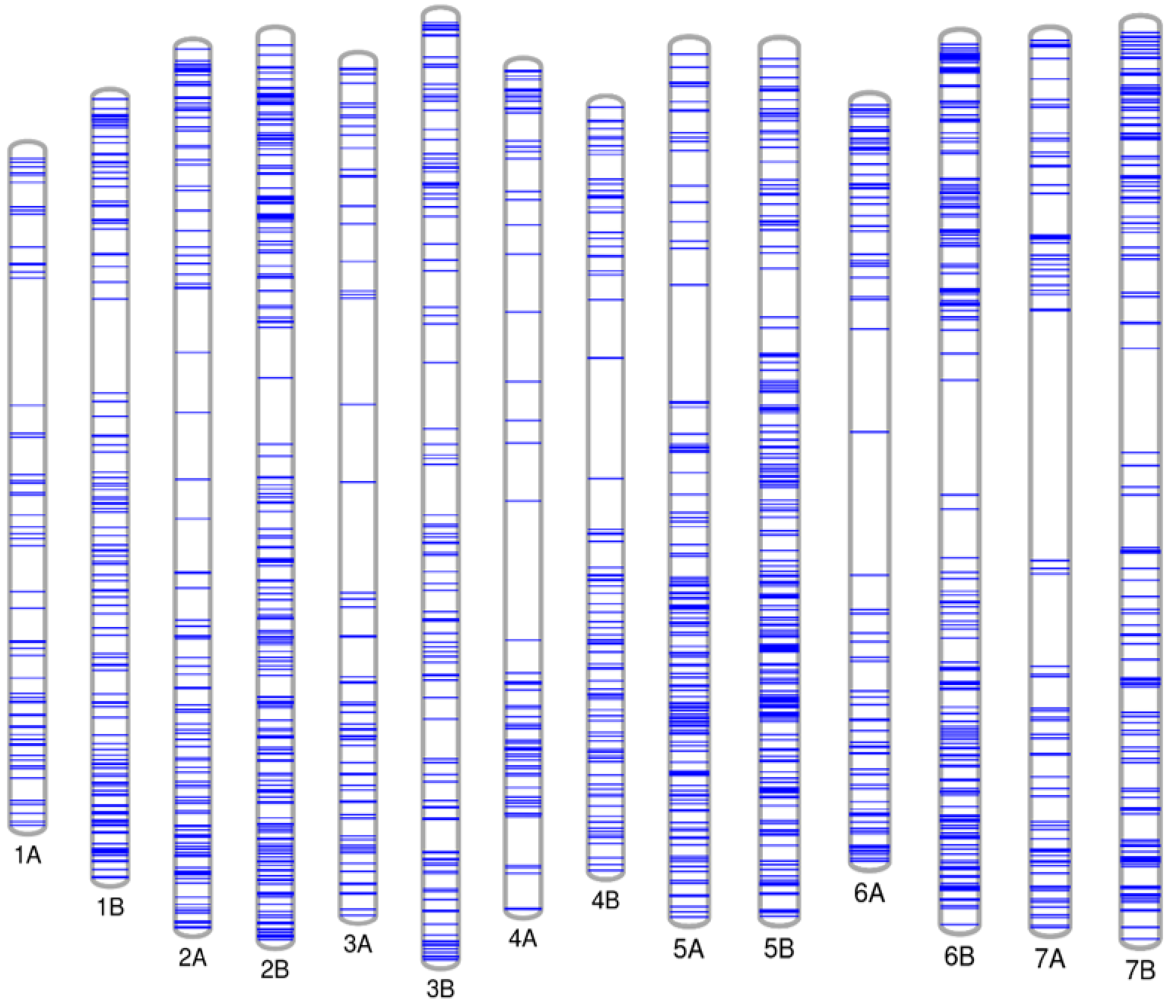

Supplementary Figure S6 Distribution of the polymorphic SNPs along the 14 chromosomes of *T. durum-Ae. speltoides* BILs based on their physical positions.

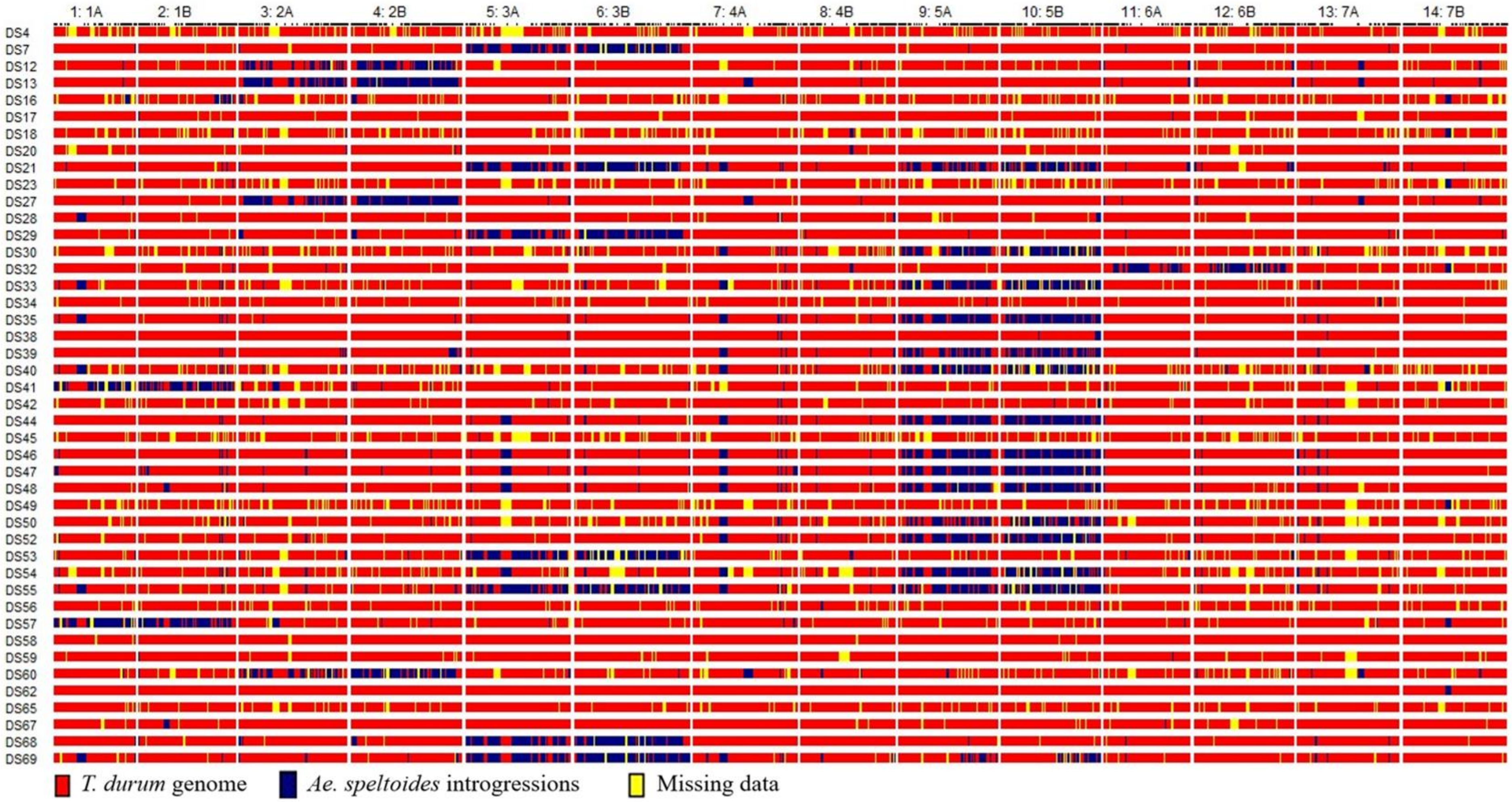
1: 1A
2: 1B
3: 2A
4: 2B
5: 3A
6: 3B
7: 4A
8: 4B
9: 5A
10: 5B
11: 6A
12: 6B
13: 7A
14: 7B
DS4
DS7
DS12
DS13
DS16
DS17
DS18
DS20
DS21
DS23
DS27
DS28
DS29
DS30
DS32
DS33
DS34
DS35
DS38
DS39
DS40
DS41
DS42
DS44
DS45
DS46
DS47
DS48
DS49
DS50
DS52
DS53
DS54
DS55
DS56
DS57
DS58
DS59
DS60
DS62
DS65
DS67
DS68
DS69
T. durum genome
Ae. speltoides introgressions
Missing data

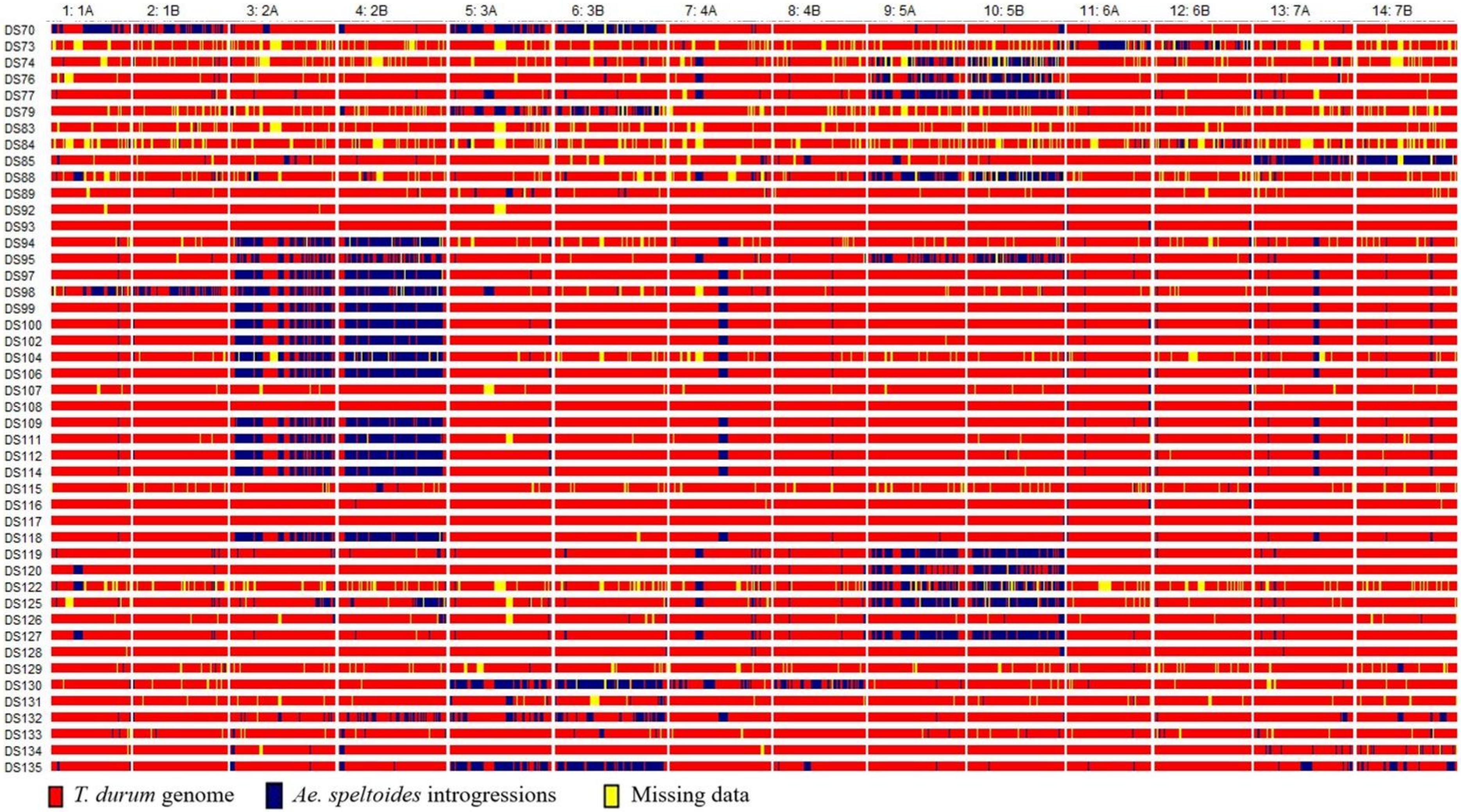
1: 1A
2: 1B
3: 2A
4: 2B
5: 3A
6: 3B
7: 4A
8: 4B
9: 5A
10: 5B
11: 6A
12: 6B
13: 7A
14: 7B
DS70
DS73
DS74
DS76
DS77
DS79
DS83
DS84
DS85
DS88
DS89
DS92
DS93
DS94
DS95
DS97
DS98
DS99
DS100
DS102
DS104
DS106
DS107
DS108
DS109
DS111
DS112
DS114
DS115
DS116
DS117
DS118
DS119
DS120
DS122
DS125
DS126
DS127
DS128
DS129
DS130
DS131
DS132
DS133
DS134
DS135
T. durum genome
Ae. speltoides introgressions
Missing data

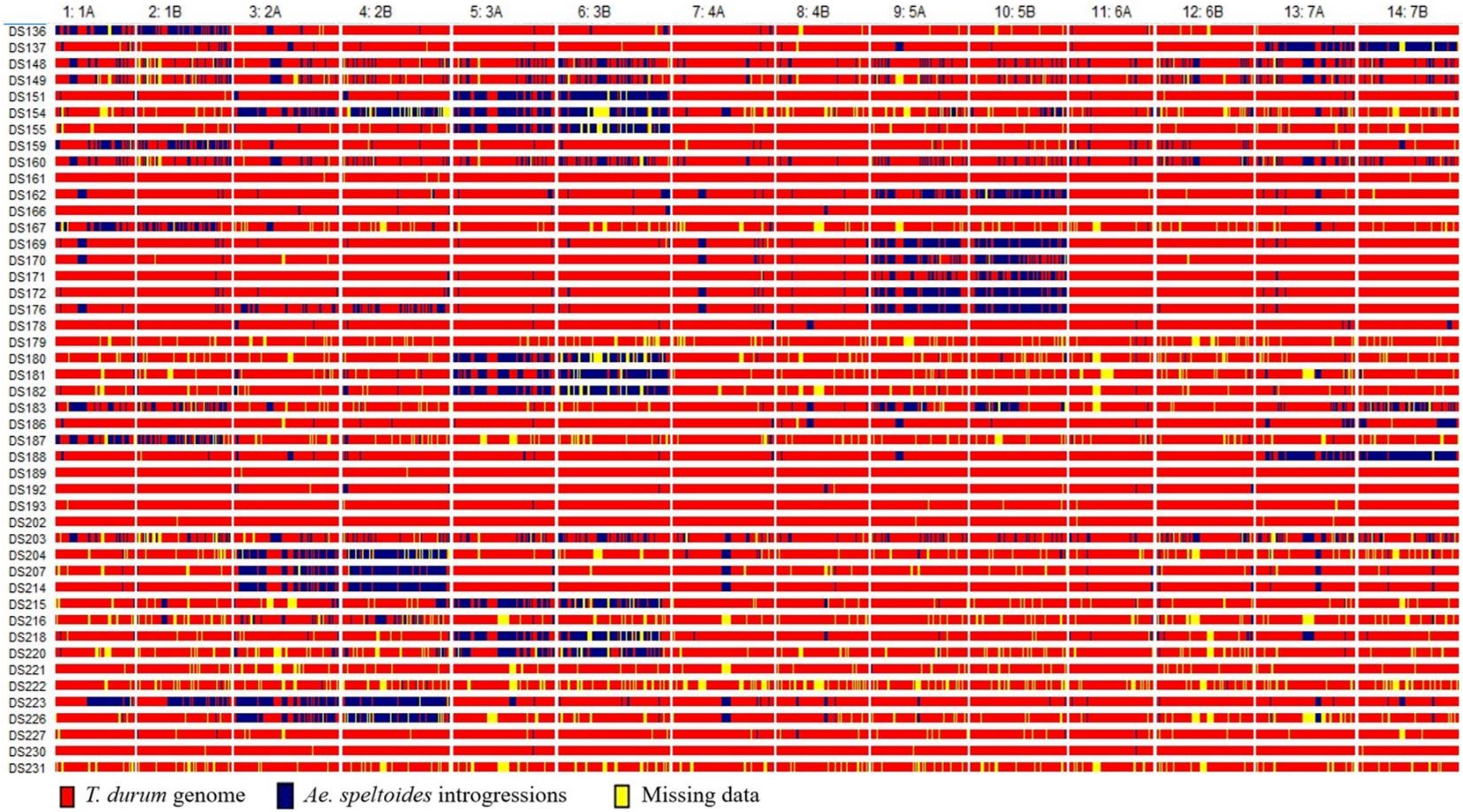
1: 1A
2: 1B
3: 2A
4: 2B
5: 3A
6: 3B
7: 4A
8: 4B
9: 5A
10: 5B
11: 6A
12: 6B
13: 7A
14: 7B
DS136
DS137
DS148
DS149
DS151
DS154
DS155
DS159
DS160
DS161
DS162
DS166
DS167
DS169
DS170
DS171
DS172
DS176
DS178
DS179
DS180
DS181
DS182
DS183
DS186
DS187
DS188
DS189
DS192
DS193
DS202
DS203
DS204
DS207
DS214
DS215
DS216
DS218
DS220
DS221
DS222
DS223
DS226
DS227
DS230
DS231
T. durum genome
Ae. speltoides introgressions
Missing data

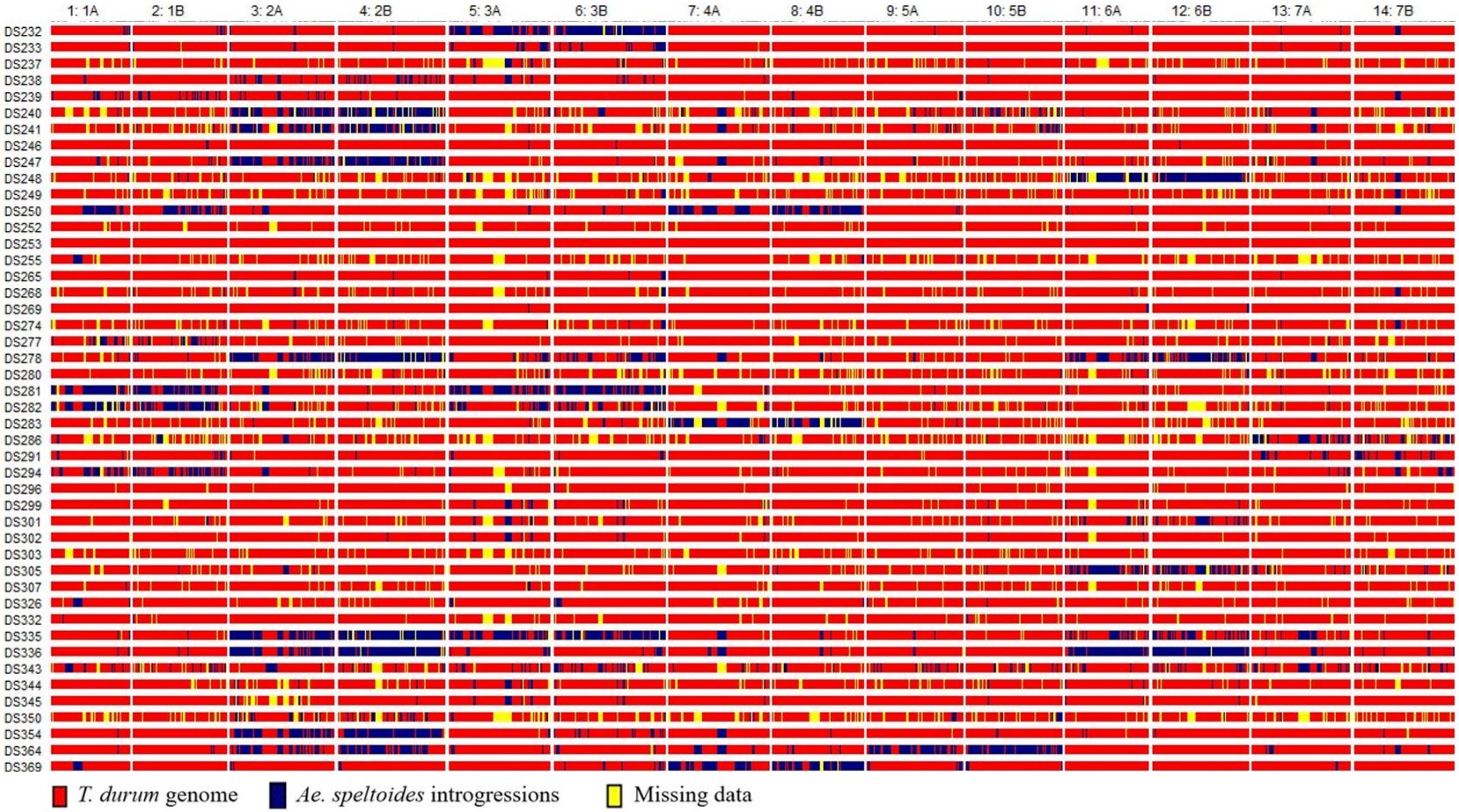
1: 1A
2: 1B
3: 2A
4: 2B
5: 3A
6: 3B
7: 4A
8: 4B
9: 5A
10: 5B
11: 6A
12: 6B
13: 7A
14: 7B
DS232
DS233
DS237
DS238
DS239
DS240
DS241
DS246
DS247
DS248
DS249
DS250
DS252
DS253
DS255
DS265
DS268
DS269
DS274
DS277
DS278
DS280
DS281
DS282
DS283
DS286
DS291
DS294
DS296
DS299
DS301
DS302
DS303
DS305
DS307
DS326
DS332
DS335
DS336
DS343
DS344
DS345
DS350
DS354
DS364
DS369
T. durum genome
Ae. speltoides introgressions
Missing data

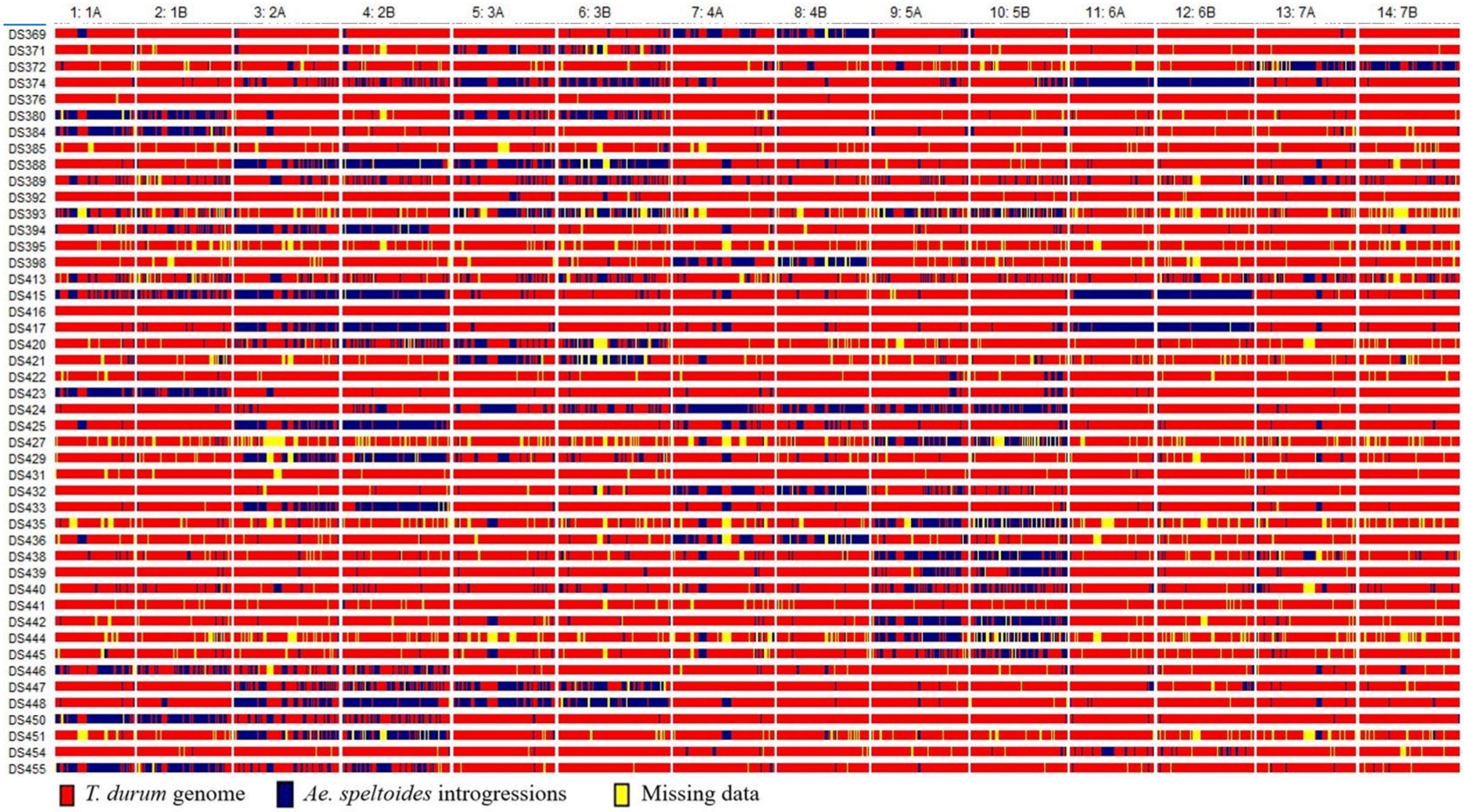

Supplementary Figure S7 Introgression profiling of the *T. durum*-*Ae. speltoides* BILs showing the *Ae. speltoides* introgressions.

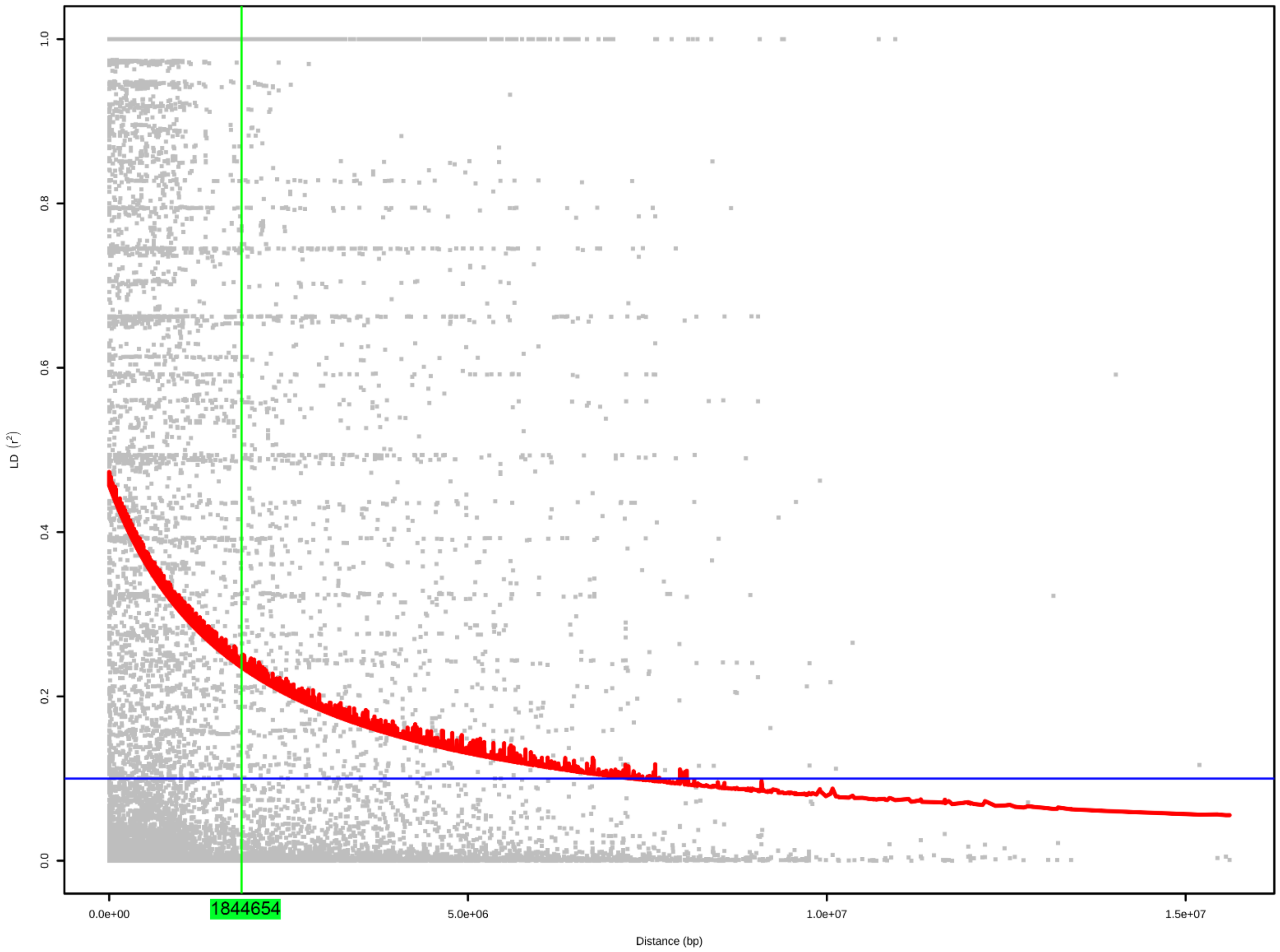

Supplementary Figure S8 Genome wide LD decay plot.

Supplementary Table S1 Mean square values of the Levene's test for the homogeneity of variances of phenotypic traits between the years 2017-18 and 2018-19

Environment	Source	df	PY	TGW	GPS	SN	SL	PL	DTH	DTM	GFD	NDVI_H
Optimum	Year	1	2.21E+09	1299.1	750.7	5.41	1.22	30.08	244.4	11.33	94.61	0.00014**
Heat-stressed	Year	1	1.37E+09	225.1	29.09	11.3	0.009	6.48	0.24	166.2	79.16	0.000033

df: degrees of freedom; **Significant at p<0.01
